# Novel Vascularized Human Liver Organoids for Modeling Alcohol‐Induced Liver Injury and Developing Hepatoprotective Therapy

**DOI:** 10.1002/advs.202511169

**Published:** 2025-12-05

**Authors:** Kangdi Yang, Xiayan Chu, Xuerui Wang, Wenkun Zhang, Jinnuo Lu, Chuting Xu, Shoucheng Hu, Guoyu Pan, Chih‐Tsung Yang, Xiaohui Zhang, Shaojin Li, Zhaobin Guo, Hanyang Liu, Guangbo Ge

**Affiliations:** ^1^ State Key Laboratory of Discovery and Utilization of Functional Components in Traditional Chinese Medicine Shanghai Frontiers Science Center of TCM Chemical Biology Institute of Interdisciplinary Integrative Medicine Research Shanghai University of Traditional Chinese Medicine Shanghai 201203 China; ^2^ Shanghai Institute of Materia Medica Chinese Academy of Sciences Shanghai 201203 China; ^3^ Future Industries Institute University of South Australia Mawson Lakes Campus Adelaide SA 5095 Australia; ^4^ State Key Laboratory of Natural and Biomimetic Drugs School of Pharmaceutical Sciences Peking University Beijing 100191 China; ^5^ Department of Hepatology and Gastroenterology Campus Virchow‐ Klinikum and Campus Charité Mitte Charité – Universitätsmedizin Berlin 13353 Berlin Germany

**Keywords:** alcoholic liver injury (ALI), drug screening, liver organoids, organoid therapy, vascularization

## Abstract

Liver organoids have emerged as transformative tools for disease modeling and therapy development. However, their inability to form functional multivascular networks significantly limits their capacity to replicate the multifaceted functions of the native human liver with high fidelity. Here, novel vascularized liver organoids (3HLOs) are constructed by co‐culturing robust human reprogrammed hepatocyte‐like cells (hrHLs) with endothelial cells (HUVECs) and mesenchymal stem cells (HUMSCs) in a 3D system. After optimization, 3HLOs form vascular architecture featuring CD31^+^ endothelial networks and CK19^+^ biliary ducts, while demonstrating enhanced hepatic function, including upregulated glycogen storage, elevated albumin secretion, accelerated indocyanine green uptake, and improved rhodamine‐123 excretion. To improve physiological relevance, 3HLOs‐on‐chip models are developed for modeling alcohol‐induced liver injury (ALI) pathogenesis and testing hepatoprotective agents. The in vitro findings closely match in vivo observations, confirming the high physiological relevance of 3HLOs‐on‐chip ALI models. Subcutaneous implantation of 3HLOs significantly attenuated liver injury and promoted hepatic regeneration in end‐stage ALI mice, primarily through establishing functional anastomoses with host microvasculature. Multi‐omics analysis revealed that 3HLOs secreted human hepatoprotective proteins into the host circulating system, thereby modulating inflammatory responses and lipid metabolism pathways. Collectively, this work offers a robust and reliable platform for ALI modeling, drug testing, and liver regeneration.

## Graphical abstract

This study successfully engineered vascularized liver organoids (3HLOs) by co‐culturing human reprogrammed hepatocyte‐like cells (hrHLs) with human umbilical vein endothelial cells (HUVECs) and human umbilical mesenchymal stem cells (HUMSCs). Upon implantation, the 3HLOs established functional vascular anastomosis with the host circulation and significantly alleviated alcohol‐induced liver injury by upregulating specific protective proteins, demonstrating considerable therapeutic potential.

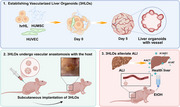

## 1 Introduction

Liver organoids are emerging as transformative tools for disease modeling and therapeutic development in hepatology.^[^ ^1^ ^]^ As 3D micro‐liver models, liver organoids offer a physiologically relevant and scalable platform that bridges the gap between basic study and clinical translation, offering transformative opportunities for disease modeling, drug discovery, personalized therapies, and liver repair.^[^ ^2^ ^]^ In the past few decades, various liver organoids derived from human primary hepatocytes (HPHs) and induced pluripotent stem cells (iPSCs) have been widely used to model liver diseases, assess hepatotoxicity, and advance drug discovery.^[^ ^3^, ^4^ ^]^ However, unlike the native liver, most liver organoids lack functional multivascular networks, significantly limiting their ability to fully replicate the complex architecture and multifaceted functions of the human liver.^[^ ^5^ ^]^

Different from other organs, the liver features a sophisticated multivascular architecture that is essential for efficient substance exchange and metabolic function. This unique structural complexity consists of intricate, hierarchically organized vascular networks, including the central vein, portal vein, hepatic artery, sinusoids, bile canaliculi, and microvilli, setting the liver apart from other organs.^[^ ^6^, ^7^ ^]^ Such a complex vascular system is crucial for supporting the multifaceted functions of the liver, including metabolism, detoxification, homeostasis, chemical exchange, and signaling transduction among different organs.^[^ ^8^, ^9^ ^]^ Although significant progress has been made in developing human liver organoids, the lack of functional multivascular networks remains a critical limitation in replicating the multifaceted functions of the human liver.^[^ ^10^ ^]^ This deficiency substantially impairs essential physiological processes, including substance exchange, metabolic activity, cellular signaling, and organoid maturation. To address these issues, there is an urgent need to develop more physiologically relevant vascularized liver organoids through innovative bioengineering strategies.

The previously established liver organoid models fail to replicate the human liver's sophisticated vascular architecture and physiological functionality, primarily due to the lack of essential non‐parenchymal cells (NPCs) or insufficient hepatocyte‐NPC interactions.^[^ ^11^ ^]^ In native human liver tissue, NPCs constitute ≈30% of liver cells and play pivotal roles in maintaining core hepatic functions.^[^ ^12^ ^]^ NPC deficiency leads to downregulation of a range of functional proteins (including cytochrome P450 enzymes, transporters, and albumin), as well as impairment of essential metabolic processes, particularly glycogen storage and overall hepatic metabolic activity.^[^ ^13^, ^14^, ^15^ ^]^ Addressing these issues requires an optimized co‐culture system harboring both hepatocytes and NPCs, which is essential to replicate the intricate architecture and functionality of the human liver.^[^ ^16^ ^]^ Unfortunately, HPHs are highly fragile under culture conditions, while iPSC‐derived hepatocyte‐like cells (HLCs) require specialized growth factors and complex induction conditions. These constraints severely hinder the establishment of functional cellular crosstalk between either HPHs or iPSCs with the essential NPCs, including Liver Sinusoidal Endothelial Cells (LSECs) and Hepatic Stellate Cell (HSCs).^[^ ^11^ ^]^

Recently, human reprogrammed hepatocyte‐like cells (hrHLs), a type of functionally mature liver‐like cells, are generated through reprogramming techniques, offering significant potential for liver disease modeling, drug screening, and regenerative medicine.^[^ ^17^, ^18^ ^]^ Unlike HPHs and the hepatocyte‐like cells derived from iPSCs, hrHLs demonstrated canonical liver functions without requiring costly and prolonged cytokine induction.^[^ ^19^ ^]^ The stable expression of hepatic transcription factors in hrHLs obviates the requirement for specialized growth factors or complex differentiation protocols, thereby enabling robust co‐culture systems with NPCs that support both vascular network formation and essential hepatic functions.^[^ ^20^, ^21^ ^]^ Moreover, the lack of pluripotent markers in hrHLs substantially reduces their tumorigenic potential, offering a safer option for regenerative therapy.^[^ ^22^, ^23^ ^]^

Herein, we report a novel vascularized liver organoid model (3HLOs) by co‐culturing hrHLs with two essential non‐parenchymal cells (HUVECs and HUMSCs). Following optimization of co‐culture conditions, the 3HLOs developed a complex, physiologically relevant vascular architecture comprising both CD31^+^ vascular networks and CK19^+^ biliary structures. The newly engineered vascularized liver organoids showed impressively enhanced hepatic functionality, demonstrating upregulated expression of key metabolic enzymes, elevated albumin secretion, accelerated indocyanine green (ICG) uptake, and improved rhodamine‐123 (Rho‐123) excretion. Notably, the functional multivascular networks in 3HLOs promoted efficient anastomosis with host micro‐vasculature, thereby establishing a perfusable blood supply.^[^ ^24^ ^]^ Encouraged by these findings, a more physiologically relevant 3HLOs‐on‐chip platform was constructed for modeling alcohol‐induced liver injury (ALI) pathogenesis and testing potential hepatoprotective agents. Furthermore, the therapeutic potential and underlying mechanisms of 3HLOs as a regenerative therapy for combating ALI were also investigated.

## 2 Results

### 2.1 3HLOs Show Multivascular Architectures and Improved Liver Functions

**Figure 1:**
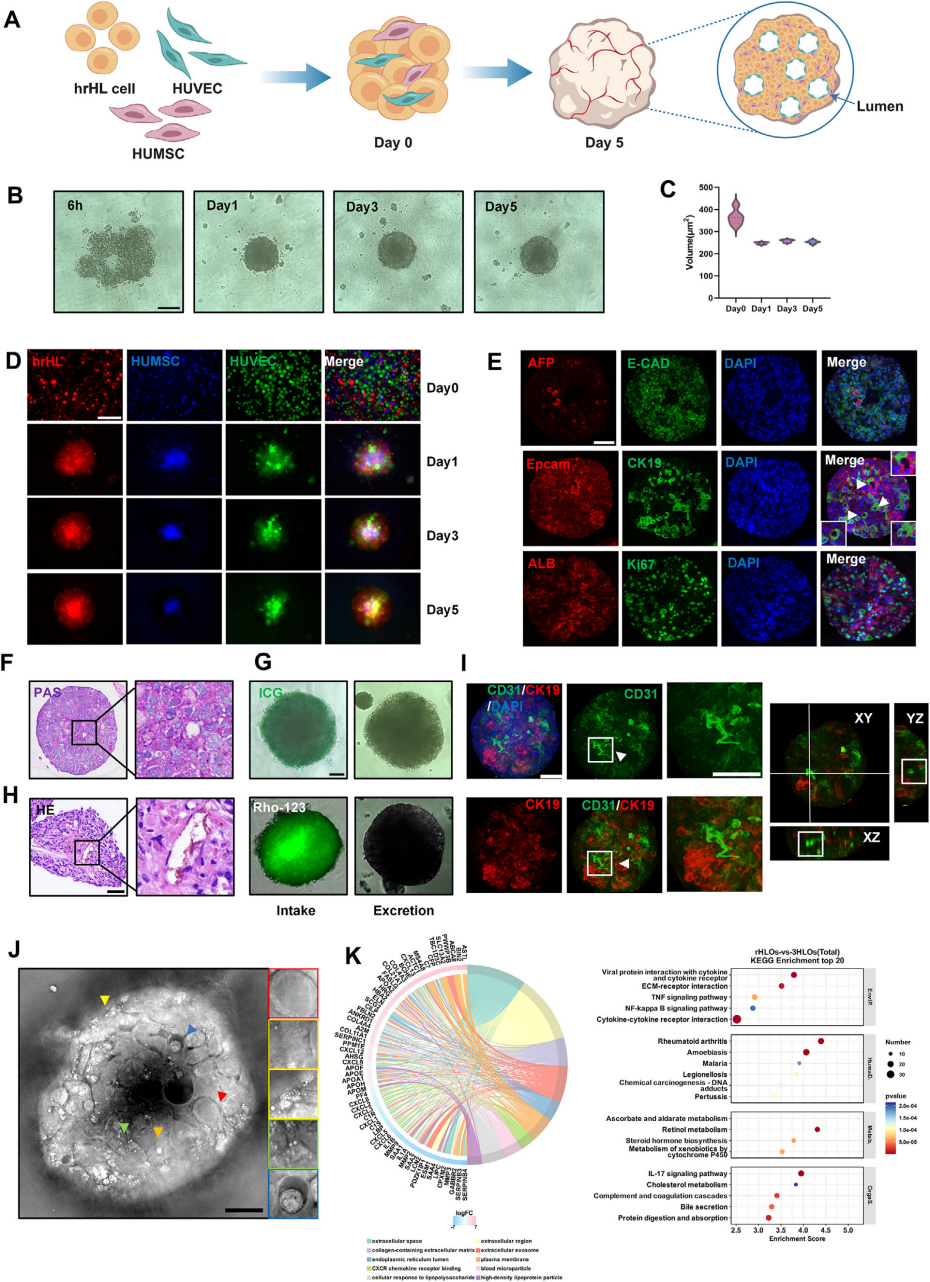
Construction, functional characterization and sequencing analysis of 3HLOs. A) Schematic illustration of the 3HLOs formation process comprising hrHL cells, HUVECs, and HUMSCs (3HLOs). Lumen structures are formed within the organoids. B,C) (B) Bright field images and (C) statistics of the projection area of the 3HLOs at 6 h, and on 1, 2, 3, and 5 days post‐3HLOs seeding. Scale bar: 50 ‌µm. D) Fluorescence images of the hrHL (red), HUMSC (blue), and HUVEC (green) with live cell tracking during 3HLOs formation. Scale bar: 50‌ µm. E) Immunofluorescence staining of 3HLOs for AFP (fetal liver marker), E‐CAD (epithelial marker), EpCAM & CK19 (intrahepatic bile duct markers), ALB (liver synthetic function marker), and Ki67 (proliferation marker). The white arrow in the second lane indicates an EpCAM & CK19 positive lumen‐like structure. Scale bar: 50 ‌µm. F) PAS Black square indicates lumen‐like structures. Scale bar: 50 ‌µm. G) Intake and excretion assay of Indocyanine green and Rho‐123 by 3HLOs. Scale bar: 50 ‌µm. H) HE staining of 3HLOs. Scale bar: 50 ‌µm. I) Immunofluorescence staining of 3HLOs for CD31 (vascular marker), CK19 (bile duct marker), and DAPI (nuclei). CD31‐positive, vascular‐like structures (indicated with white arrow) and CK19‐positive, bile duct‐like structures (indicated with white arrow) were interwoven within 3HLOs. Scale bar: 20‌ µm. J) Visualization of the structures within the 3HLOs with holotomographic microscopy. Structures (indicated with arrows) and zoom‐in in the right column indicate cell nuclei (red), mitochondria (orange), lysosomes (yellow), lipid droplets (green), and bile duct‐like structures (blue). Scale bar: 50 ‌µm. K) Chord diagram analysis (left) and dot plot analysis (right) results of transcriptomic analysis between rHLOs and 3HLOs. Data are presented as violin plots showing the median (solid line) and interquartile range (dashed lines).

Incorporating human umbilical vein endothelial cells (HUVECs) and human umbilical cord mesenchymal stem cells (HUMSCs) was hypothesized to promote vascular network formation within the hrHL organoid, enhancing the structural and functional similarities over native liver tissue. In this context, HUVECs, HUMSCs, and hrHL were co‐seeded into U‐shaped ultralow‐adhesion plates to generate tri‐culture organoids (termed 3HLOs), while control organoids containing only hrHL (rHLOs) were prepared in parallel (**Figure** **1**A; rHLO characterization in Figures S1 and S2, Supporting Information). Following 6 h of loose aggregation, 3HLOs stabilized in size over 5 days (Figure 1B,C), with progressive integration observed among various cell types over time (Figure 1D).^[^ ^24^, ^25^ ^]^

Compared to rHLOs, 3HLOs showed superior functional maturation, as evidenced by significantly reduced α‐fetoprotein (AFP) staining (Figure 1E) and elevated expression levels of hepatic maturation markers (Figure S3A, Supporting Information). Notably, unlike rHLOs, 3HLOs developed functional bile canaliculi, as demonstrated by well‐organized CK19^+^EpCAM^+^ ductal structures with well‐defined lumen‐like morphology (Figure 1E; Table S1, Supporting Information indicated by white arrows) (Figure 1E). Furthermore, functional characterization revealed a coordinated enhancement in key hepatic activities in 3HLOs compared to rHLOs, including significantly greater glycogen storage (periodic acid‐Schiff, PAS, staining; Figure 1F; Figure S3D, Supporting Information), improved uptake of indocyanine green (ICG) and the clearance of rhodamine‐123 (Rho‐123) (Figure 1G; Figure S3B,C, Supporting Information), as well as elevated albumin (Alb) synthesis rates (Figure S3A, Supporting Information). Hematoxylin and eosin (HE) staining identified lumenized structures in 3HLOs. (Figure 1H), while immunofluorescence imaging confirmed 3D vascular networks co‐expressing CD31 (endothelial) and CK19 (cholangiocyte) markers (Figure 1I; Movies S1–S3, Supporting Information). These networks exhibited greater complexity and scale than those in rHLOs. Holotomographic microscopy (HTM) resolved intracellular architectures within 3HLOs, including nuclei (red), mitochondria (orange), lipid droplets (yellow), lysosomes (green), and luminal spaces (blue arrows) (Figure 1J). These findings highlight substantial hepatocyte–NPC interactions in 3HLOs, which greatly facilitate both hepatic morphogenesis and functional maturation.

To further characterize the differences between rHLOs and 3HLOs, transcriptomic analyses and differential metabolite analyses were performed. Transcriptomic analysis revealed that cholesterol metabolism, xenobiotic metabolism (e.g., P450 enzymes), protein digestion/absorption, bile secretion, and angiogenesis (Figure 1K) were profoundly enriched in 3HLOs compared to those of rHLOs. Interestingly, the mRNA levels of key drug‐metabolizing enzymes (including CYP1A1, CYP2E1, and CYP3A4) and bilirubin glucuronidation enzyme (UGT1A1) in 3HLOs were significantly higher than those in rHLOs (Figure S3E,F and Table S2, Supporting Information). These findings suggest that 3HLOs possess improved metabolic functions. Metabolomic analysis further revealed enhanced endogenous metabolic activity in 3HLOs (Figure S3G, Supporting Information).

Notably, the key transcriptional factors that suppress angiogenesis (e.g., HEY1, CCN1, VASH1, VASH2) are downregulated in 3HLOs (Figure S3H,I, Supporting Information), further supporting the enhanced potential of 3HLOs for vascular network formation. Our attempts to integrate hepatic stellate cells and immune cells into rHLOs (termed hSMOs) resulted in poorly organized aggregates with peripheral cell dispersion rather than proper incorporation (Figure S4A–D, Supporting Information), indicative of weak cell‐cell adhesion/interactions. Transcriptomic analysis of hSMOs showed no enrichment in lipid/cholesterol metabolism, xenobiotic detoxification, or glycogen storage pathways (Figure S4F,G, Supporting Information), reinforcing the unique efficacy of the HUVEC‐HUMSC‐hrHL triad in driving functional vascularization and hepatic maturation.

### 2.2 Functional Anastomosis is Formed within 3HLOs

**Figure 2:**
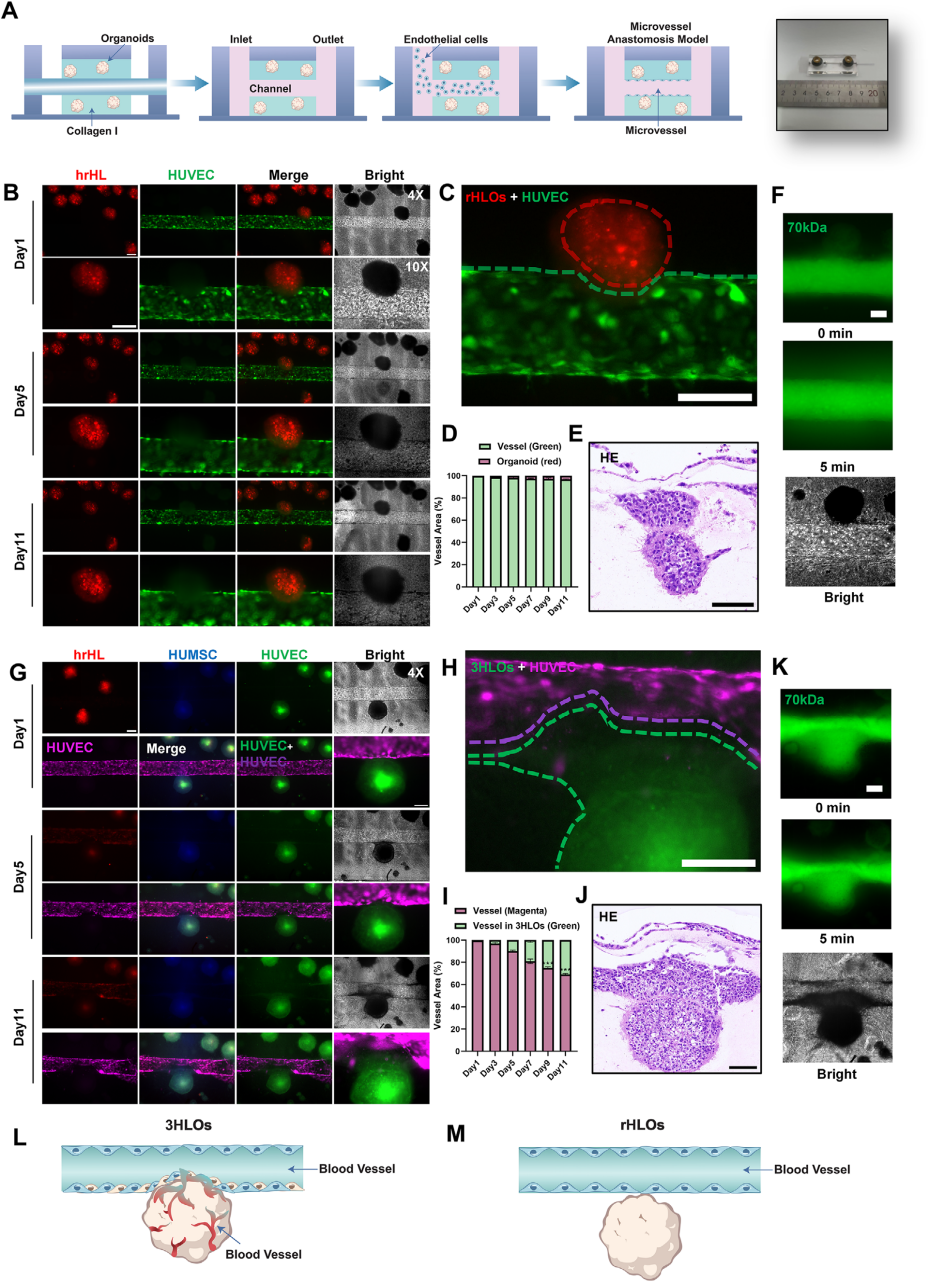
Functional anastomosis is formed in 3HLOs‐ rather than in rHLOs‐on‐Chip. A) Schematic illustration of the liver organoid‐on‐chip fabrication process. B) Fluorescence images of interactions between rHLOs (red) in the collagen hydrogel and HUVECs (green) in the main channel vessel of the microfluidic devices. Images in the upper lane were observed with 4× objective lens and the lower lane with 10×. Scale bar: 50 ‌µm. C) Magnification image of rHLOs (red)‐microvessel (green) contact sites on Day 11 to visualize the interaction between rHLOs in the collagen hydrogel and HUVECs in the main channel vessel. Scale bar: 50 ‌µm. D) Statistics of rHLOs (red) and endothelial cells (green) accounting for the proportion of a fixed area (200 × 200 ‌µm^2^, n = 5). E) HE staining of collagen gels pulled out from the organoid‐on‐chip. The gel contains rHLOs and an endothelialized main channel vessel, the site organoid in proximity to the main channel vessel is focused in order to examine the interaction between the main channel vessel and organoid. Scale bar: 50 µm. F) Fluorescence images of rHLOs‐main channel vessel contact sites (n = 9) after perfusing FITC‐labeled 70 kDa dextran (fluorescent dye) into the main channel vessel. Images were taken immediately (upper) and 5 min (middle) after the dye being perfused into the main channel vessel. A bright‐field image (below) was taken simultaneously to visualize the proximity between the organoid and the main channel vessel. Scale bar: 50 ‌µm. G) Fluorescence images of interaction between 3HLOs (hrHL: red, HUMSCs: blue, HUVECs: green) and HUVECs (magenta) in the main channel vessel of the microfluidic device from Day 1 to Day 11. Images in the upper lane were observed with 4 × objective lens and the lower lane with 10 ×. Scale bar: 50‌ µm, n = 9. H) Magnification image of 3HLOs microvessel contact site on Day 11 HUVEC in the 3HLOs is labeled as green, and in the main channel vessel as magenta. Scale bar: 50‌ µm, n = 16. I) Statistics of 3HLOs (green) and endothelial cells (magenta) accounting for the proportion of a fixed area (200 × 200 ‌µm^2^, n = 5). J) HE staining of collagen gel pulled out from organoid‐on‐chip. The site organoid in proximity to the main channel vessel is focused in order to examine the interaction between the main channel vessel and the organoid. Scale bar: 50 µm, n = 16. K) Fluorescence images of 3HLOs‐main channel vessel contact site after perfusing FITC‐labeled 70 kDa dextran (fluorescent dye) into the main channel vessel. Images were taken immediately (upper) and 5 min (middle) after the dye being perfused into the main channel vessel. A bright field image (lower) was taken simultaneously to visualize the proximity between the organoid and the main channel vessel. Scale bar: 50 ‌µm, n = 9. L) Illustration of the contact site between 3HLOs and the main channel. Endothelial cells in the 3HLOs extending into the main channel vessel, while endothelial cells in the main channel vessel wrap the organoids. M) Illustration of the contact site between rHLOs and the main channel vessel interaction between rHLOs and the main channel vessel is rarely observed. Data are expressed as the mean ± standard deviation (SD).

To further enhance the physiological relevance of 3HLOs, a customized microfluidic organoids‐on‐chip (OOC) platform was developed for constructing 3HLOs‐on‐chip models. As depicted in **Figure** **2**A, the model featured an endothelialized central channel (termed “microvessel”) alongside liver organoids embedded in type I collagen, designed to assess functional anastomoses between the engineered microvessel (HUVEC in green) (Figure 2B) and the intra‐organoid vasculature. Time‐lapse monitoring of rHLOs over 11 days (Figure 2B) revealed no significant structural or functional interactions between the rHLOs (red) and the microvessel at the interface (Figure 2C). Minimal structural integration between the organoids and the microvessel was observed throughout the experimental period. Quantification of HUVEC monolayer integrity within the channel remained consistently high (>98%) over time (Figure 2D), further supporting the absence of functional anastomosis between rHLOs and the microvessel. Histological analysis via HE staining at the interface confirmed limited physical interaction between rHLOs and the microvessel channel (Figure 2E). To rigorously evaluate rHLO‐microvessel anastomosis, we performed perfusion of FITC‐conjugated 70 kDa dextran through the central channel. A weak fluorescence signal was detected in rHLOs (Figure 2F; Movies S4, Supporting Information), demonstrating a lack of functional rHLO‐microvessel anastomosis.

In contrast, 3HLOs demonstrated progressive structural integration with the microvessels when growing in the OOC. HUVECs (green) originating from the 3HLOs migrated toward and enveloped the microvessel (magenta) at the interface (Figure 2G), with this structural interconnection expanding over time (Figure 2H). Quantitative analysis confirmed the time‐dependent expansion of the 3HLO‐microvessel interconnection, calculated as the proportion of green fluorescence (3HLO‐derived HUVECs) occupying the magenta‐labeled main channel vasculature within the defined region (Figure 2I). These results were corroborated by HE staining, which highlighted extensive structural integration between the 3HLOs and the microvessel (Figure 2J). Notably, immediate perfusion of 3HLO with minimal leakage was observed following FITC‐conjugated 70 kDa dextran flowing through the microvessel, indicating a robust anastomosis formed without undermining barrier functionality of the microvessel. (Figure 2K; Movies S5, Supporting Information).

Notably, no significant anastomosis was observed in hSMOs. Instead of forming functional anastomosis, hSMOs induced vascular erosion, creating severe structural discontinuities in the engineered vessels (Figure S4H,I, Supporting Information). HE staining revealed dispersed cells at the periphery of hSMOs, indicative of compromised vascular integrity (Figure S4E, Supporting Information). This disruptive phenotype may be regulated by cytokines from immune and stellate cells within hSMOs, potentially destabilizing both organoid and vascular architectures. These observations suggest that HUVECs and HUMSCs regulated vasculogenesis within the 3HLOs are critical for establishing functional anastomoses with pre‐existing vasculature in the organoid‐on‐chip platform (Figure 2L). Conversely, organoids lacking these components (e.g., rHLOs and hSMOs) exhibited no physiologically relevant vascular integration and failed to form functional anastomosis with existing vasculature (Figure 2M).

### 2.3 Functional Anastomosis Formed In Vivo After Implanting Vascularized 3HLOs

**Figure 3:**
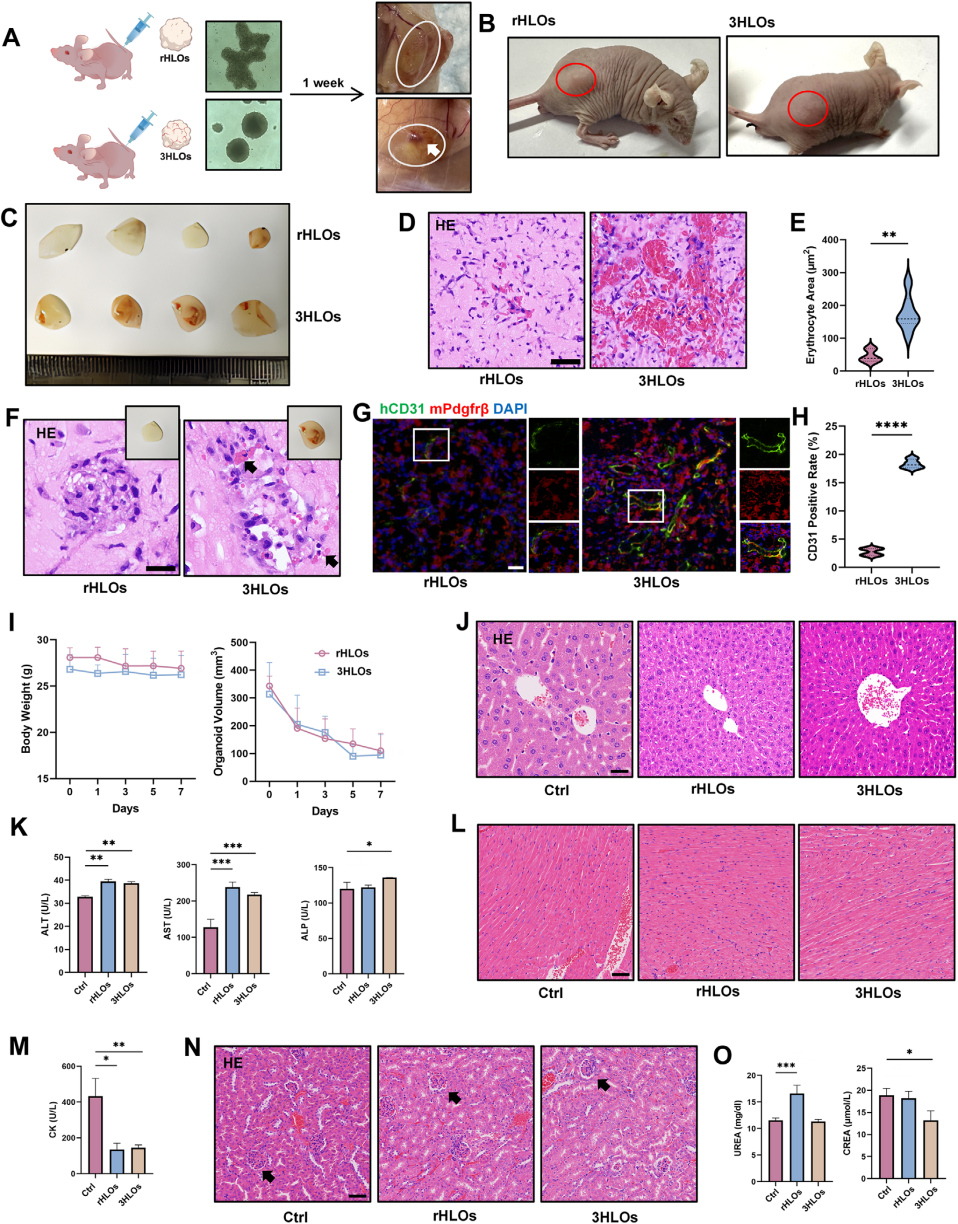
Functional anastomosis formed in 3HLOs transplanted in nude mice. A) Schematic illustration of the timeline that 3H‐ or rHLOs subcutaneously transplanted into nude mice. The white arrow indicates the formation of blood vessels in the 3HLOss (lower image), which is not observed in the rHLOs (upper image). B) Photographs of nude mice that are subcutaneously transplanted 3HLOsor rHLOs. Red circles indicate the organoid transplantation sites. n = 5. C) Photographs of the rHL‐ or 3HLOs harvested from nude mice 10 days after transplantation to observe the blood vessel distribution. n = 5. D–F) HE staining (D), quantification (n = 8, (E), and magnified HE staining (F) images of the 3HLOs‐ and rHLOs harvested from nude mice 10 days after transplantation. Red blood cells (red region) are widely distributed in 3HLOs. Scale bar: 50 ‌µm (D) and 20 µm (F). G,H) hCD31 & mPdgfrβ IF staining (G) and statistics of positive rate (H) of the 3HLOs and rHLOs harvested from nude mice 10 days after transplantation. Scale bar: 50 µm, ^****^ *p* < 0.0001. I) Statistical analysis of weight changes in nude mice after transplanting 3HLOs‐ and rHLOs (left) and organoid volume change (right, volume = long diameter ^*^ short diameter^2^/2). ^*^ *p* < 0.05. J–O) HE staining of the liver (J), heart (L) kidney (N), and serologic examination of liver (K), heart (M), and kidney (O) injury markers from nude mice after transplanting rHLOs, 3HLOs, or vehicle control for 10 days. Black arrows indicate intact glomeruli without visible focal points in Figure O. n = 8, ns *p* > 0.05; ^*^ *p* < 0.05; ^***^ *p* < 0.001, Scale bar: 50 µm. Data are expressed as the mean ± SD. Data are presented as violin plots showing the median (solid line) and interquartile range (dashed lines).

To further evaluate the potential of 3HLOs in establishing anastomoses and facilitating blood supply, we subcutaneously implanted 3HLOs into the right posterior flank of nude mice for a 7‐day period, along with rHLOs as a control (**Figure** **3**A). Both rHLOs and 3HLOs formed visible protrusions at the implantation site, indicating efficient subcutaneous implantation with minimal structural dissipation for each organoid type (Figure 3B). Comparative analysis of harvested organoids revealed significantly denser vascular networks (red) within 3HLOs compared to rHLOs (Figure 3C). Histological exploration via HE staining revealed a markedly higher density of red blood cells (RBCs) in 3HLOs compared to rHLOs (Figure 3D). Quantitative analyses confirmed a 2.89‐fold increase in RBC counts within 3HLOs relative to rHLOs (Figure 3E). High‐resolution HE imaging further identified distinct RBC clusters in implanted 3HLOs (Figure 3F, indicated by black arrows), a feature absent in rHLOs. Immunohistochemical (IHC) analysis demonstrated well‐developed vascular networks (CD31^+^) in 3HLOs, contrasting sharply with the complete lack of comparable structures in rHLOs. Quantification of hCD31‐positive areas indicated 9.66‐fold higher vasculatures presence in 3HLOs compared to the rHLOs (Figure 3G,H). As shown in Figure 3G, structural integration of the 3HLO vasculature with the host system was demonstrated by co‐staining of hCD31 and mPdgfrβ (vascular markers in the mouse microvasculature). These findings clearly demonstrate that the vascularized 3HLOs (human) can form functional anastomoses with host microvessels (mouse), thereby facilitating blood supply to the implanted organoids. Of note, these findings are consistent with the results observed in OOC models.

### 2.4 Biosafety Profiling of 3HLOs‐Implantation in Mice

Next, the safety profiles of subcutaneous implanting 3HLOs or rHLOs were comprehensively investigated. Neither 3HLOs nor rHLOs caused significant alterations in host body weight (Figure 3I, **left**) or showed signs of uncontrolled growth. As shown in Figure 3I (right), both organoid types demonstrated progressive volume reduction over time. Critically, histological and biochemical analyses revealed no evidence of liver (Figure S5I, Supporting Information), cardiac, renal (Figure 3J–O), or pulmonary injury (Figure S5K, Supporting Information) post‐transplantation. Additionally, blood glucose levels remained stable across all experimental groups (Figure S5J, Supporting Information), further supporting the safety profile of the implanted organoids. To elucidate the molecular mechanisms underlying the observed differences between 3HLOs and rHLOs following subcutaneous implantation, RNA sequencing was performed. Principal component analysis (PCA) revealed a distinct segregation between two organoid types (Figure S5A, Supporting Information). In line, the top significant DEG further demonstrates distinct patterns in biological processes and molecular functions (Figure S5B,C, Supporting Information). Differential gene expression analysis identified significant extracellular matrix‐related alterations in rHLOs (Figure S5D, Supporting Information), suggesting active cellular remodeling following subcutaneous implantation. In contrast, 3HLOs displayed enhanced immune‐related activity, characterized by upregulation of Spn and Tnf (implicated in leukocyte recruitment) and activation of IL‐12 signaling pathways (Figure S5E,F, Supporting Information). Additionally, 3HLOs exhibited elevated nicotinamide adenine dinucleotide (NADH) metabolic activity (Figure S5G,H, Supporting Information), a finding with potential relevance for modeling alcohol‐induced liver injury. These in vivo results demonstrate that subcutaneous implantation of 3HLOs shows safety profiles, which inspire us to further investigate the therapeutic potential of 3HLOs as a novel regenerative therapy.

### 2.5 Modeling Alcoholic Liver Injury Using Vascularized 3HLOs and Organoid‐On‐Chip

**Figure 4:**
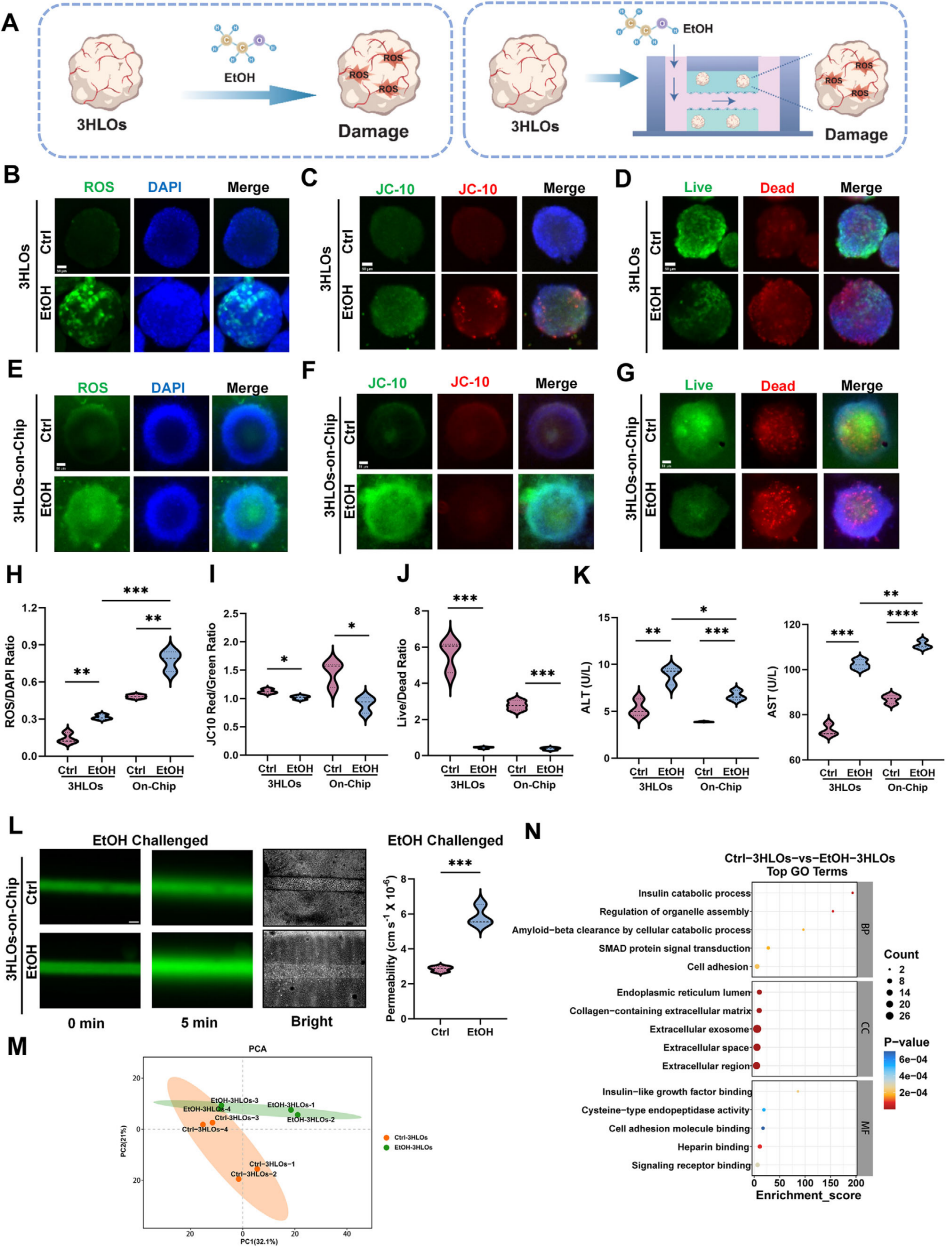
Construction of an alcoholic liver injury model using 3HLOs and microfluidic chip technology. A) Schematic illustration of ALI model using 3HLOs (left) and 3HLOs‐on‐a‐chip (right) B–D) Fluorescence images of DCFH‐DA (ROS probe) (B), JC‐10 (mitochondrial membrane potential probe) (C), Calcein‐AM (live cell: green, dead cell: red) (D) labeled 3HLOss with (EtOH group) or without (Ctrl group) ethanol treatment. Scale bar:50 ‌µm. E–G) Fluorescence images of DCFH‐DA (E), JC‐10 (F), and Calcein‐AM (G) labeled 3HLOs‐on‐chip with (EtOH group) or without (Ctrl group) ethanol treatment. Scale bar:50 µm. H–J) Statistics of ROS/DAPI ratio, JC‐10Red/JC‐10Green ratio, and Live‐Green/Dead‐Red cell ratio changes in Ctrl group and EtOH group in 3HLOs (left) and 3HLOs‐on‐a‐chip (right). n = 9. ^*^ *p* < 0.05; ^**^ *p* < 0.01; ^***^ *p* < 0.001; ^****^ *p* < 0.0001. K) Detection of ALT and AST in the supernatant of 3HLOs (left) and 3HLOs‐on‐chips (right) with (EtOH group) or without ethanol (Ctrl group) treatment. n = 9. ns P >0.05; ^*^ *p* < 0.05. L) Fluorescence images (left) and permeability statistics (right) of the main channel vessel in 3HLOs‐on‐chips model with (EtOH group) or without (Ctrl group) ethanol treatment after injecting 70 kDa into the main channel vessel. Calculation of 70 kDa penetration using the Poisson equation. n = 5. ns *P* > 0.05; ^*^ *p* < 0.05. Scale bar:5 ‌µm. M,N) PCA and GO protein metabolism analysis in EtOH and Ctrl groups. n = 4. Data are presented as violin plots showing the median (solid line) and interquartile range (dashed lines).

Encouraged by the enhanced biomimetic properties, 3HLOs were then adapted to establish a physiologically relevant in vitro model of alcoholic liver injury (ALI) via incorporating quantitative assessment parameters (**Figure** **4**A,B,H). The 3HLOs were divided into an untreated control (Ctrl) and ethanol (EtOH)‐exposed group, while the key ALI hallmarks were tested following 72 h of treatment. Quantitative analysis revealed significantly elevated ROS levels in the EtOH‐exposed group compared to Ctrl (Figure 4B,H left), suggesting alcohol‐induced oxidative stress. Consistently, EtOH‐exposed 3HLOs showed pronounced mitochondrial depolarization, as demonstrated by increased green fluorescence intensity (Figure 4C,I left). Furthermore, EtOH‐exposed 3HLOs appeared substantial cell death (indicated by red fluorescence) when compared to Ctrl (Figure 4D,J **left**).

To evaluate hepatocellular damage in the ALI model, we quantified classic liver injury hallmarks in the 3HLO culture supernatant, including alanine aminotransferase (ALT) and aspartate aminotransferase (AST). Consistent with clinical manifestations of alcohol‐induced hepatotoxicity, EtOH‐exposed 3HLOs exhibited significantly elevated ALT and AST levels compared to untreated controls. These results robustly validate the utility of 3HLOs for modeling ALI with high physiological fidelity.

Following oral ingestion, alcohol rapidly crosses the intestinal barrier and enters systemic circulation, and then reaches the liver through hepatic portal circulation. To recapitulate both systemic alcohol transport and hepatic pathophysiology, we adapted the aforementioned 3HLOs‐on‐chip platform where EtOH was delivered through the biomimetic microvessel. This dynamic perfusion system modeled the hepatic microenvironment to simulate both early pathogenesis and progressive ALI dynamics. After 72 h of EtOH exposure, ROS, JC‐10 staining, and cellular viability (live/dead assay) were tested in both control and EtOH groups (Figure 4E–G). As shown in Figure 4H–K (**right**), EtOH‐exposed 3HLOs exhibited significantly elevated ROS levels, dysfunctional mitochondrial membrane potential (measured by JC‐10), and increased cell death compared to controls. Comparative analysis in Figure S6A–C,G (Supporting Information) demonstrated the enhanced pathological relevance of modeling ALI using the 3HLOs‐on‐chip platform compared to conventional organoid‐only models. The integrated vascular architecture of the OOC system enabled real‐time observation of alcohol effects on vasculature. Perfusion with 70 kDa FITC‐dextran through the microvessel revealed EtOH‐induced vasculature dysfunction, as evidenced by undermined barrier integrity in EtOH groups. Bright‐field imaging further revealed densely packed and continuous vascular structures in the Ctrl group, in contrast to the sparse and disrupted organization observed in the EtOH‐challegned group (Figure 4L). Quantitative assessment of 70 kDa FITC‐dextran permeability revealed significantly elevated vascular leakage in EtOH groups compared to controls. Concurrently, proteomic profiling of perfusates was performed from 3HLOs‐on‐chip with and without EtOH exposure. Substantial differentially expressed proteins (DEPs) were identified (Figure 4M), with significant enrichment in GO cellular components such as collagen‐containing extracellular matrix, extracellular exosomes, and extracellular space (Figure 4N). These results suggested EtOH exposure remarkably changed the exocrine behaviors of 3HLOs, such as exosome production. In addition, rHLO and the associated OOC model (rHLO‐on‐chip) were adapted for ALI modeling (Figures S6D–F,H and S7A–N, Supporting Information), with results comparable to the 3HLO counterparts. These findings collectively outline the capabilities of 3HLOs‐on‐chip in recapitulating the pathogenesis and progression of ALI.

### 2.6 3HLOs‐On‐Chip ALI Model Shows High In Vivo Consistency for Evaluating Hepatoprotective Agents

**Figure 5:**
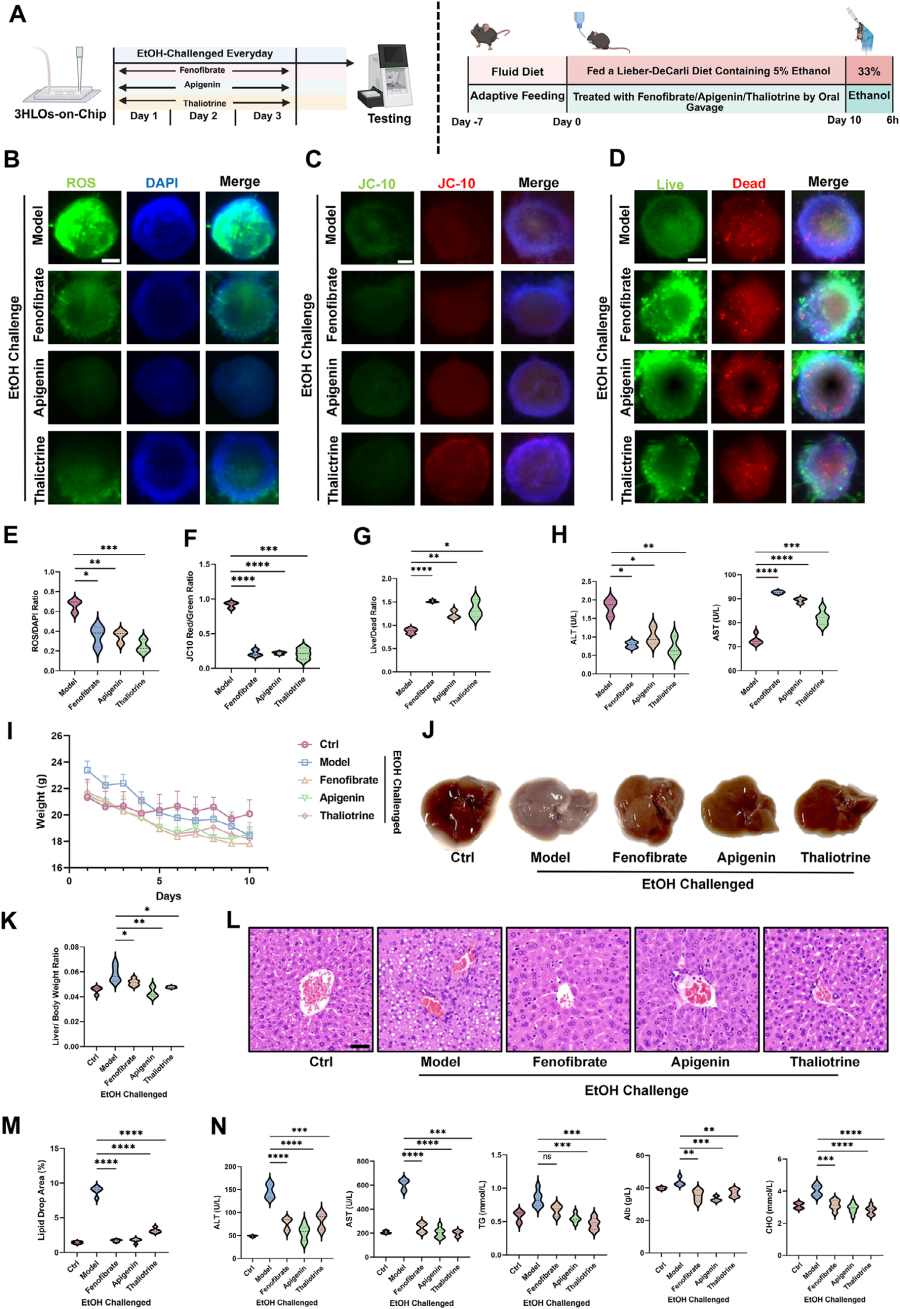
Alcoholic liver injury 3HLOs‐on‐chips model for evaluating hepatoprotective agents. A) Flowchart of Drug Screening Using 3HLOs‐on‐Chip and Animal Models. B–D) Fluorescence images of DCFH‐DA (B), JC‐10 (C), or Calcein‐AM (D) labeled 3HLOs‐on‐chips under ethanol exposure (model), or fenofibrate, apigenin, or thaliotrine treatment under ethanol exposure. Scale bar: 50 ‌µm. E–H) Statistics of ROS/DAPI ratio (E), JC‐10 Red/JC‐10 Green ratio (F), live‐green/dead‐red cell ratio (G,H), and ALT and AST in supernatant in ethanol exposure (model), fenofibrate, apigenin, or thaliotrine treatment groups. n = 9. ^*^ *p* < 0.05; ^**^ *p* < 0.01; ^***^ *p* < 0.001; ^****^ *p* < 0.0001. I–K) Body weight changes (I), liver appearance (J), and liver‐to‐body weight ratio (K) in C57BL/6J mice for the Sham Ctrl group, model group, fenofibrate group, apigenin group, and thaliotrine group. n = 8. ^*^ *p* < 0.05; ^**^ *p* < 0.01. L,M) HE staining (K) and quantification of lipid droplet area percentage changes (L) of liver tissues among the model, fenofibrate, apigenin, and thaliotrine groups. n = 8. ^****^ *p* < 0.0001, Scale bar: 50‌ µm. N) Serum biochemical analysis of liver function indicators (ALT, AST, TG, Alb, CHO) of the model, fenofibrate, apigenin, and thaliotrine groups. ns *p* > 0.05; ^**^ *p* < 0.01; ^***^ *p* < 0.001; ^****^ *p* < 0.0001. Data are expressed as the mean ± SD. Data are presented as violin plots showing the median (solid line) and interquartile range (dashed lines).

Given that 3HLOs‐on‐chip can mimic the onset and progression of ALI, the OOC model was then used to test anti‐ALI agents. An anti‐oxidative agent (apigenin) and an anti‐inflammatory compound (thaliotrine) were selected for further tests.^[^ ^26^, ^27^ ^]^ Meanwhile, fenofibrate, a marketed peroxisome proliferator‐activated receptor α (PPARα) agonist, was also used as a positive hepatoprotective agent.^[^ ^28^ ^]^ Day 1 of the ALI model was defined as the starting point for drug treatment (**Figure** **5**A). Following the protocols and quantitative indicators established in the previous section, we assessed ROS, JC‐10, and cell viability as indicators for hepatoprotective efficacy (Figure 5B–D). The results showed that fenofibrate, apigenin, and thaliotrine significantly reduced ROS accumulation (Figure 5E), alleviated mitochondrial dysfunction (Figure 5F), and improved hepatocyte viability (Figure 5G) to similar extents. Notably, thaliotrine showed more potent anti‐oxidative activity when compared to fenofibrate, while both apigenin and thaliotrine exhibited superior effects in enhancing hepatocyte viability. We also measured ALT and AST levels in the perfusates and observed in downregulation as well (Figure 5H). The in vivo relevance of the 3HLOs‐on‐chip model was evaluated using a well‐established ALI mouse model (National Institute on Alcohol Abuse and Alcoholism, NIAAA protocol).^[^ ^29^ ^]^ As shown in Figure 5I, the mice receiving fenofibrate, apigenin, or thaliotrine showed less severe weight loss compared to those in the ALI model group. Liver morphology assessments revealed that apigenin and thaliotrine restored liver appearance to levels comparable to the sham Ctrl group, demonstrating similar efficacy to fenofibrate (Figure 5J). The liver‐to‐body weight ratio, a critical indicator of liver swelling, was significantly reduced in the apigenin‐ and thaliotrine‐treated groups compared to the ALI model group, with their effects surpassing those of fenofibrate (Figure 5K). Furthermore, HE staining showed substantial mitigation of lipid droplet accumulation in the livers of the apigenin and thaliotrine groups, comparable to the fenofibrate group (Figure 5L,M). Serum biochemical analyses indicated that levels of ALT, AST, triglycerides (TG), ALB, cholesterol (CHO), and other ALI‐associated markers were significantly downregulated following treatment with apigenin or thaliotrine, demonstrating efficacy similar to fenofibrate (Figure 5N).

The results from both the 3HLOs‐on‐chip ALI models and ALI mouse models demonstrate that apigenin and thaliotrine effectively mitigate liver injury, showing substantial hepatoprotective effects comparable to or superior to the positive agent fenofibrate. The consistent therapeutic outcomes across these models highlight the strong physiological relevance of the 3HLOs‐on‐chip system and its potential as a biomimetic platform for studying ALI pathogenesis and testing hepatoprotective agents.

### 2.7 3HLOs Significantly Mitigate Alcohol‐Induced Liver Damage In Vivo

**Figure 6:**
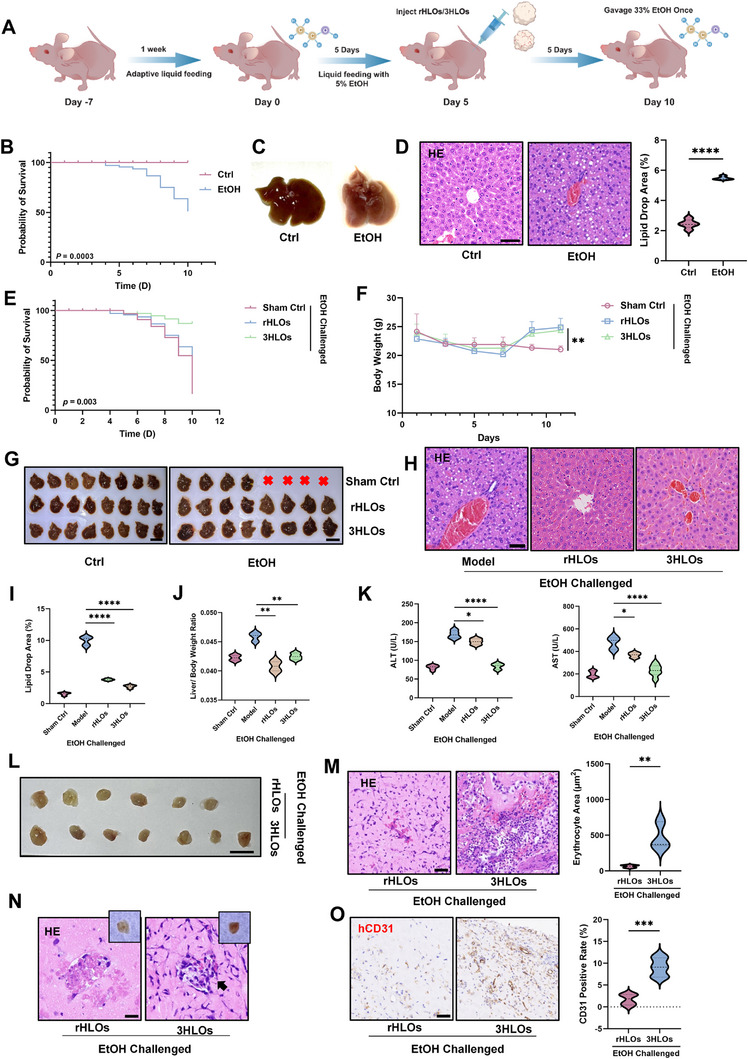
The therapeutic effects of 3HLOs for mitigating alcoholic liver injury in ALI mice. A) Schematic illustration of the construction of alcoholic liver injury mice. B) Survival curves for the mice with (EtOH) or without (Ctrl) alcohol diet. n = 10. *P* = 0.0003. C) Photography of liver appearance on the 5^th^ day with an alcohol diet following sacrificing the mice. D) HE staining to assess lipid droplet formation in the liver of alcohol‐exposed mice (left) and statistics of lipid droplet area (right). n = 5. ^****^ *p* < 0.0001. Scale bar:50 ‌µm. E–J) Survival (E) and body weight (F) change curves of the alcohol‐exposed mice transplanted with rHLOs, 3HLOs, or sham control (sham ctrl). ^*^ *p* < 0.05. G–J) Liver appearance (G), HE staining (H), and quantification (I) of the liver lipid droplet area (I) and liver‐to‐body weight ratios (J) from alcohol‐exposed mice transplanted with rHLOs, 3HLOs, or sham control. n = 8. Scale bar:50 ‌µm.^****^ *p* < 0.0001. K) Serum biochemical analysis of liver function indicators ALT (left), AST (right) from alcohol‐exposed mice transplanted with rHLOs, 3HLOs, or sham control. n = 8. ^*^ *p* < 0.05; ^****^ *p* <0.0001. L) Photography of rHLOs and 3HLOs harvested from alcohol‐exposed mice. M,N) HE staining (M) and magnified image (N) of rHLOs and 3HLOs harvested from alcohol‐exposed mice, abundant red blood cells are observed in the 3HLOs (left), and quantification of red blood cell area in organoid samples from left images (right)(n≥5). ^**^ *p* < 0.01. Scale bar:50 ‌µm. O) IHC staining of CD31 for the transplanted organoids (left) and quantification of CD31 positive rates (right). ^***^ *p* < 0.001. n = 5. Scale bar: 50 ‌µm. Data are expressed as the mean ± SD. Data are presented as violin plots showing the median (solid line) and interquartile range (dashed lines).

Next, the therapeutic potential of 3HLOs for mitigating ALI and ALI‐associated liver failure (**Figure** **6**A) was investigated. To optimize transplantation timing, we tracked disease progression in EtOH‐exposed nude mice, noting mortality onset beginning on day 5 post‐EtOH‐challenged (Figure 6B). Concurrently, gross morphological changes in liver tissue (Figure 6C) and histopathological analysis revealed significant hepatic steatosis, characterized by microvesicular and macrovesicular lipid droplet accumulation (Figure 6D), confirming the transition to liver injury by day 5. To evaluate therapeutic efficacy, we performed subcutaneous transplantation of 3HLOs, rHLOs, or controls on day 5 post‐EtOH exposure. Survival analysis demonstrated significant mortality reduction in both 3HLO‐ and rHLO‐transplanted groups compared to sham controls (Figure 6E). Strikingly, 3HLO transplantation achieved a final mortality rate of ≈25%, twofold improvement over rHLOs (≈50%) and threefold over untreated animals (≈90%). Furthermore, 3HLO recipients exhibited marked body weight recovery relative to sham controls (Figure 6F), underscoring their superior therapeutic potential. HE demonstrated that 3HLO transplantation markedly reduced hepatic lipid accumulation compared to rHLOs, with quantitative assessment confirming its superior efficacy (Figure 6G–I). Mice receiving 3HLOs also exhibited normalized liver‐to‐body weight ratios, approaching levels observed in controls (Figure 6J). Serum biomarker analysis revealed significant reductions in ALT and AST levels in 3HLO‐treated animals, restoring values to sham control baselines, whereas rHLO transplantation showed no comparable improvement (Figure 6K). Crucially, 3HLO‐transplanted mice developed robust vascular networks under EtOH stress, contrasting sharply with the minimal vasculature observed in rHLO recipients (Figure 6L). HE staining of 3HLO transplantation sites demonstrated abundant erythrocyte infiltration (Figure 6M,N, **black arrows**), indicating the formation of functional anastomoses with the host vasculature system. By contrast, erythrocyte infiltration or functional anastomoses were rarely detected in rHLOs. IHC quantification further confirmed the enhanced multivascular architectures in 3HLOs, with CD31‐positive areas significantly exceeding those in rHLOs (Figure 6O). These findings indicate that 3HLOs transplantation effectively rescues alcohol‐induced liver failure. The superior treatment outcomes of 3HLOs underscore the critical role of engineered vascularization in treating ALI, positioning 3HLOs as a promising pro‐regenerative strategy for end‐stage liver pathologies.

### 2.8 Multi‐Omics Analysis Reveals the Therapeutic Mechanisms of 3HLOs

**Figure 7:**
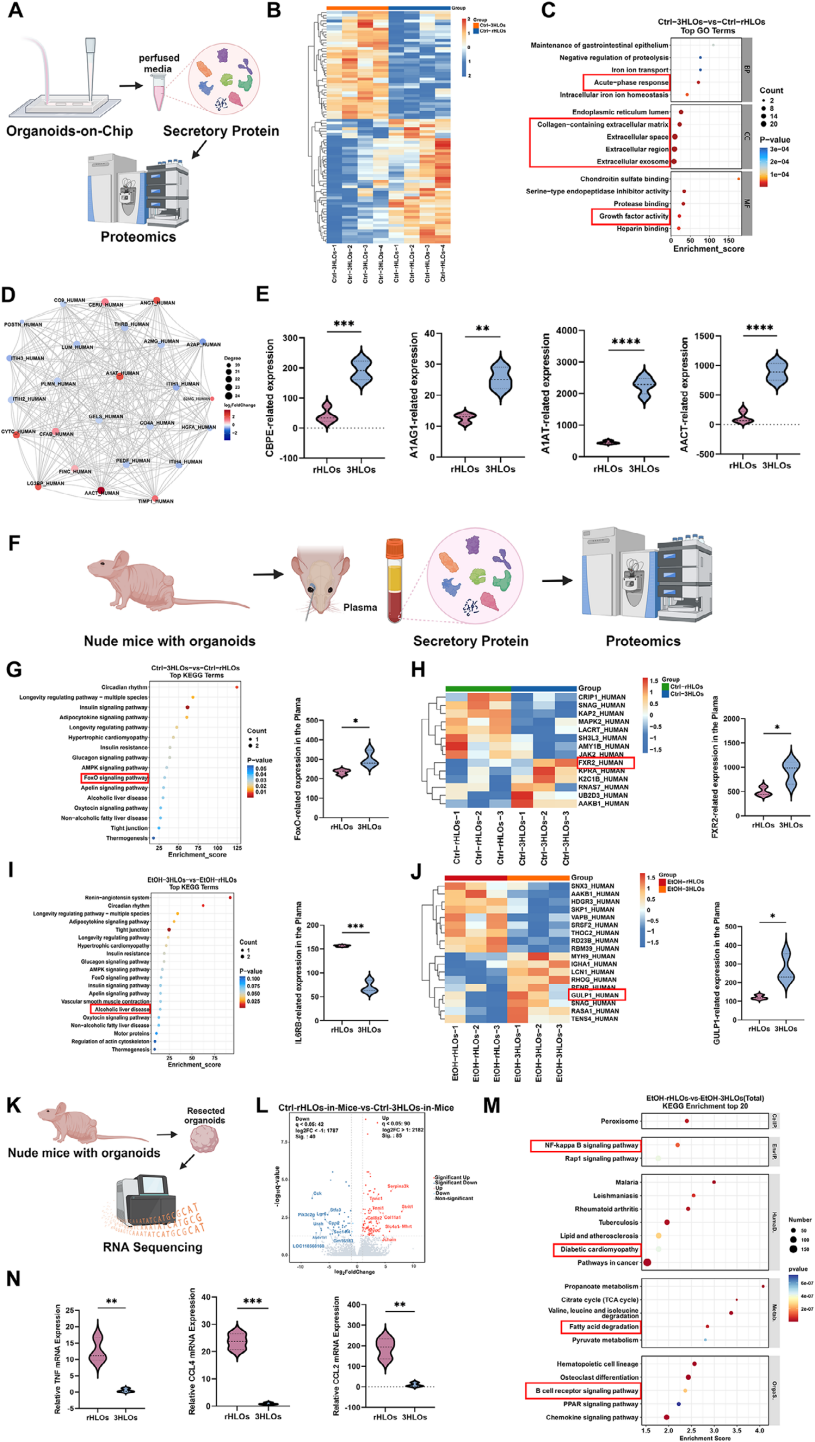
Investigating the mechanisms of 3HLOs for combating ALI. A) Schematic diagram of proteomics. (Design adapted from biorender.com). B–D) Volcano plot (B), bubble plot (C), and PPI (D) network of proteomic differences in the chip under non‐alcoholic conditions. E) Detection of protein changes in A1AT, A1AG, CBEP, and AACT within the chip. n = 5 ^**^ *p* < 0.01; ^***^ *p* < 0.001; ^****^ *p* < 0.0001. F) Schematic diagram of plasma proteomics in mice. (Design adapted from biorender.com). G) KEGG analysis of human‐derived proteins in plasma from mice loaded with different organoids under non‐alcoholic conditions (left). FoxO protein levels in plasma (right). n = 5. ^*^ *p* < 0.05. H) Heatmap of human‐derived proteins in plasma from mice loaded with different organoids under non‐alcoholic conditions (left). FXR2 protein levels in plasma (right). n = 5 ^*^ *p* < 0.05. I) KEGG analysis of human‐derived proteins in plasma from mice loaded with different organoids in the alcoholic model (left). ILRB protein levels in plasma (right). n = 5. ^***^ *p* < 0.001. J) Heatmap of human‐derived proteins in plasma from mice loaded with different organoids in the alcoholic model (left). GULP1 protein levels in plasma (right). n = 5. ^*^ *p* < 0.05. K) Schematic diagram of transcriptomics (Design adapted from biorender.com). L) Volcano plot of transcriptomic analysis for two types of organoid samples under non‐alcoholic conditions. M) KEGG analysis of two organoid types in the alcoholic model. N) The mRNA levels of TNF‐, CCL4‐, and CCL2 ‐related RNAs in two organoids following alcohol treatment. n = 5. ^**^ *p* < 0.01; ^***^ *p* < 0.001. Data are expressed as the mean ±SD. Data are presented as violin plots showing the median (solid line) and interquartile range (dashed lines).

As described above, 3HLOs created functional anastomoses with host micro‐vessels and demonstrated superior therapeutic efficacy for combating end‐stage ALI when compared to rHLOs, which encouraged us to further explore the underlying theraputic mechanisms. To this end, the secreted proteome from both 3HLOs and rHLOs was analyzed first (**Figure** **7**A). As shown in Figure 7B, quantitative proteomic analysis of OOC perfusate after 72 h revealed 86 types secretome signatures between 3HLOs and rHLOs. GO enrichment analysis highlighted the differential secreted proteins, which are tightly associated with acute‐phase response pathways, extracellular matrix (ECM) remodeling, exosomal cargo, and growth factor signaling (Figure 7C). Protein‐protein interaction network (PPIN) analysis identified alpha‐1‐antitrypsin (A1AT) and alpha‐1‐antichymotrypsin (AACT) as central hubs within the 3HLO secretome, with A1AT exhibiting the highest network centrality—a hallmark of functional importance (Figure 7D). Consistently, plasma proteomics showed that the levels of three acute‐phase proteins (A1AT, AACT, A1AG1) were significantly elevated in 3HLO‐transplanted mice compared to rHLO recipients (Figure 7E).

To further elucidate therapeutic mechanisms of 3HLOs, the human proteome in the plasma from nude mice transplanted with 3HLOs or rHLOs was comprehensively analyzed (Figure 7F). Proteomic profiling demonstrated a marked enrichment of FoxO signaling pathway constituents, critical molecular regulators mediating alcohol‐induced hepatic inflammation, in 3HLO‐treated murine models compared to their control counterparts. (Figure 7G). These findings aligned with the markedly elevated protein levels of FoxO in 3HLO‐transplanted mice, suggesting that the FoxO signaling was significantly activated. Pathway enrichment analysis revealed that 3HLOs therapy promopted bile acid homeostasis (e.g., FXR2) and lipid metabolism while suppressing lipogenic pathways (Figure 7H). In alcohol‐challenged mice, 3HLOs transplantation downregulated interleukin‐6 receptor expression and of ALI‐associated markers (Figure 7I), while elevating the levels of autophagy and oxidative stress response proteins (e.g., GULP1) (Figure 7J). These results suggest that 3HLOs mitigate ALI progression in vivo by regulating inflammatory pathways and endogenous metabolism.

Transcriptome analysis of both alcohol‐exposed 3HLOs and alcohol‐exposed rHLOs revealed distinct inflammatory dynamics (Figure 7K,L). Function enrichment analysis demonstrated significant downregulation of pro‐inflammatory pathways in 3HLOs, including NF‐κB signaling and B‐cell receptor signaling (Figure 7M). Simultaneously, lipid metabolism pathogenesis pathways, linking to atherosclerosis metabolism and fatty acid degradation, were suppressed. As shown in Figure 7N, mRNA quantitative analysis confirmed the mRNA levels of inflammatory mediators (TNF, CCL4, CCL2) in 3HLOs were significantly reduced compared to rHLOs following long‐term alcohol exposure. These findings clearly demonstrate that compared to rHLOs, 3HLOs show better host compatibility and anti‐inflammatory activity.

Taken together, 3HLOs mitigate alcohol‐induced liver damage primarily through the release of acute‐phase proteins (such as A1AT and AACT) into the host circulating system. These proteins act as hepatoprotective factors for ameliorating alcohol‐induced inflammatory responses, thereby alleviating ALI pathological progress, such as lipid accumulation in the liver and other organs.

## 3 Discussion

In this study, we developed a novel liver organoids model featuring enriched vascular and bile canaliculi structures by co‐culturing human‐induced hepatocytes (hrHLs), endothelial cells (HUVECs), and human umbilical cord mesenchymal stem cells (HUMSCs). The primary rationale for selecting these cell types was based on practical feasibility, robust functionality, and clinical translation potential. Currently, primary human liver NPCs, including LSECs and HSCs, are challenging to obtain in large quantities from human liver tissue, meanwhile are highly prone to dedifferentiation and loss of specialized functions during in vitro culture. In contrast, HUVECs and HUMSCs are commercially available with minimal batch‐to‐batch variation. As multipotent stem cells, HUMSCs exhibit diverse differentiation capabilities. Increasing studies have demonstrated that HUMSCs can be induced to express hepatocyte‐specific markers, including Albumin (Alb), through treatment with nicotinamide and Activin A, among other factors.^[^ ^30^, ^31^ ^]^ This vascularized humanized organoid demonstrated significantly enhanced hepatic functions, including nutrient transport, glycogen storage, biosynthesis, and metabolic activity. Both in vivo and organoid‐on‐chip models revealed that the engineered vasculature within the organoid established functional anastomoses with host blood vessels, allowing for a stable nutrient and oxygen supply. This vascular integration facilitated essential advantages for modeling ALI and evaluating therapeutic efficacy. Notably, the newly developed vascularized organoids (3HLOs) demonstrated significantly enhanced efficacy in mitigating alcohol‐induced liver failure compared to hepatocyte‐only organoids (rHLOs).

The hrHLs were generated by reprogramming fibroblasts using key transcription factors (FOXA3, HNF1A, HNF4A) critical for hepatocyte differentiation.^[^ ^32^, ^33^ ^]^ Co‐culture with HUMSCs and HUVECs further promoted hepatocyte maturation, as evidenced by upregulated Alb expression, downregulated α‐fetoprotein levels, and enhanced secretion of acute‐phase proteins. These findings align with previous studies that vascularization improves oxygen/nutrient delivery and facilitates paracrine signaling, thereby accelerating the structural and functional maturation of liver organoids.^[^ ^34^, ^35^ ^]^ In fact, vascularization has remained a central challenge in tissue engineering and regenerative medicine. Vascularization fuels oxygen supply, nutrient delivery, and clearance of metabolic waste through the engineered vascular networks, in line with guiding the orderly differentiation and functional maturation of parenchymal cells via growth factors secreted by vascular endothelial cells and cell‐cell interactions.^[^ ^36^, ^37^, ^38^ ^]^ These coordinated biological processes collectively enhance the physiological activity and tissue homeostasis of engineered tissues. In addition, prior to full vascular maturation, endothelial cells and hepatic vascular niches interacted closely with hepatocytes via paracrine signaling or physical interactions, orchestrating hepatocyte polarization and sinusoidal organization.^[^ ^39^ ^]^

Noteworthy, HUMSCs also played pivotal roles in 3HLO maturation. HUMSCs function as a “signaling hub” by secreting a spectrum of growth factors that initiate and sustain angiogenesis. Specifically, HUMSCs constitutively secrete vascular endothelial growth factor (VEGF), the primary trigger for angiogenesis. VEGF binds to the VEGFR2 receptor on HUVECs, activating downstream MAPK and PI3K‐Akt signaling pathways, which in turn promote HUVEC proliferation and tubulogenesis.^[^ ^40^, ^41^ ^]^ Concurrently, HUMSC‐derived angiopoietin‐1 (ANGPT1) engages the Tie2 receptor on HUVECs, enhancing endothelial cell interactions and collectively regulating vascular maturation. Furthermore, studies have reported that HUMSC‐secreted basic fibroblast growth factor (bFGF) directly supports hepatocyte maturation and survival, indirectly facilitating vascularization in liver organoids.^[^ ^42^ ^]^ The unique co‐culture system employed in this study enables robust physical connections among different cell types, guided by prior paracrine signaling. Immunofluorescence data from this work confirmed upregulated VE‐cadherin expression in the organoids. This adhesion molecule mediates homophilic dimerization, tightly linking adjacent endothelial cells. Additionally, CD31, abundantly expressed on HUVEC surfaces, plays a critical role in vascular morphogenesis. The U‐bottom, ultra‐low attachment culture environment further provides an extracellular matrix‐like niche conducive to vascular self‐organization and network formation. These factors contribute collectively to enhance the functional superiority of 3HLOs over rHLOs, as supported by in vivo and in vitro data.

The 3HLO model accurately recapitulated core pathological features of ALI and drug responses, which closely mirrored in vivo observations and manifested its high fidelity in modeling ALI progression.^[^ ^25^, ^43^, ^44^ ^]^ In fact, non‐parenchymal cells, particularly endothelial cells, have been extensively reported to play central roles in alcohol‐induced injury. For example, alcohol induces liver sinusoidal endothelial cell (LSEC) dysfunction via CYP2E1 upregulation, which promotes HSP90 acetylation and disrupts its interaction with endothelial nitric oxide synthase (eNOS).^[^ ^45^ ^]^ This cascade suppresses nitric oxide (NO) production, thereby triggering hepatic inflammatory responses.^[^ ^46^ ^]^ Additionally, alcohol‐induced inflammation upregulated endothelial adhesion molecules (ICAM‐1, VCAM‐1, P‐selectin) in the liver, exacerbating immune cell infiltration and hepatic damage.^[^ ^47^, ^48^ ^]^ These findings underscore the necessity of incorporating vascular components for physiologically relevant ALI modeling.

Strikingly, we found subcutaneous implantation of 3HLOs significantly improved liver repair and survival in alcohol‐injured mice compared to rHLOs. Proteomic profiling further demonstrated that 3HLOs exhibited significantly elevated release of acute‐phase proteins, including A1AT and AACT, compared to rHLOs. This enhanced secretory profile largely explains the superior therapeutic efficacy of 3HLOs against ALI. Notably, hepatocyte‐derived acute‐phase proteins like A1AT play a crucial protective role by inhibiting neutrophil‐derived proteolytic enzymes (e.g., elastase, cathepsin G), thereby preventing excessive degradation of hepatic proteins and hepatocyte injury.^[^ ^49^, ^50^, ^51^ ^]^ Additionally, these proteins attenuate inflammatory cascades by suppressing proinflammatory cytokine release, while promoting hepatocyte proliferation through Cyclin D1‐mediated cell cycle regulation, thereby facilitating hepatic regeneration.^[^ ^52^, ^53^ ^]^ Critically, the therapeutic efficacy of A1AT for mitigating acute liver failure and inflammation has been reported previously.^[^ ^54^, ^55^ ^]^ A1AT exerts its protective effects by inhibiting caspase‐3/caspase‐8 activity in liver homogenates, in turn blocks apoptotic signaling pathways. Furthermore, both serum TNF levels and TNF‐converting enzyme activity levels in ALI mouse models were significantly decreased following A1AT administration, which well‐explained our observations.^[^ ^56^, ^57^ ^]^ Of particular translational relevance, human‐derived A1AT demonstrated significant therapeutic benefits in ALI mouse models, highlighting its translational potential despite species differences. These findings demonstrate the impressive therapeutic efficacy of A1AT across species, providing mechanistic support for our findings that human organoid transplantation acts as a feasible therapy for ameliorating ALI.^[^ ^57^ ^]^

Notably, while employing the less conventional approach of subcutaneous organoid transplantation rather than through in situ or intravenous approaches, we observed robust therapeutic efficacy in our study. Traditional stem cell/organoid delivery methods predominantly involve in situ organ surgical transplantation or intravenous injection.^[^ ^58^, ^59^ ^]^ However, surgical methods are inherently invasive, and intravenous administration carries documented risks of pulmonary embolism, rapid hepatic/splenic clearance, and even mortality in clinical cases.^[^ ^60^ ^]^ In light of our data that functional anastomosis is a critical determinant underlying the preserved therapeutic activity of subcutaneously implanted organoids in hepatic repair, we propose two synergistic mechanisms: (i) Stromal cell‐mediated vascularization promotes the functional maturation of hepatocytes within organoids, enhancing their secretory capacity for ALI‐mitigating proteins such as acute‐phase reactants; (ii) Established vascular anastomoses with host circulation potentiate the hepatic delivery and bioavailability of these therapeutic proteins. The coordinated action of these mechanisms collectively underpins the superior therapeutic performance of 3HLOs over rHLOs in ameliorating alcohol‐induced liver injury.

Although 3HLOs‐on‐chip has been successfully used for modeling ALI and screening hepatoprotective compounds, several limitations require further investigation. First, while immune‐deficient murine models enable pilot organoid therapy studies, their exclusive use precludes critical assessment of human‐adaptive immune responses to xenogeneic organoids aiming for clinical translation. Second, beyond the established role of vascular integration in driving therapeutic outcomes, comprehensive characterization of transplanted organoids warrants further study, particularly their longitudinal biodistribution, metabolic fate, and differentiation trajectories. Third, while our findings in acute injury models are promising, the therapeutic durability of 3HLOs‐on‐chip must be validated in ALI paradigms. Future studies should prioritize immunocompetent humanized mouse models to evaluate immune interactions, advanced multimodal in vivo tracking to resolve organoid dynamics in real time, and longitudinal evaluations of ALI progression. These steps will bridge mechanistic insights with clinical needs, accelerating the translation of organoid‐based therapies.

## 4 Conclusion

In summary, an innovative vascularized liver organoid system (3HLOs) was engineered via assembling robust human reprogrammed hepatocyte‐like cells (hrHLs) with HUVECs and HUMSCs. Compared to control organoids (rHLOs), 3HLOs exhibit vascular and biliary luminal networks, as well as enhanced hepatic function. To further enhance the physiological relevance and functionality of 3HLOs, a customized microfluidic organoids‐on‐chip platform (3HLOs‐on‐chip) was developed. 3HLOs‐on‐chip showed the multivascular architectures and the multifaceted functions of the human liver. Compared to 2D models, the 3HLOs‐on‐chip system demonstrates enhanced cellular diversity, spatially organized architecture, and intricate blood perfusion dynamics. Both 3HLOs and 3HLOs‐on‐chip were subsequently used for modeling ALI dynamics and testing hepatoprotective agents. It was also found that 3HLOs could effectively attenuate liver injury and promote hepatic regeneration in end‐stage ALI mice, offering a novel, safe, and reliable therapeutic strategy for mitigating ALI. Collectively, this work provides a biomimetic platform with superior physiological fidelity for in‐depth investigations on liver diseases and therapies. Future work will focus on refining these models, deciphering the dynamic interactions between liver organoids and blood vessels, and further expanding the applications of this platform in drug discovery and precision therapeutics.

## 5 Experimental Section

### Animal and Cell Culture

Seven‐week‐old C57BL/6J male mice and Balb/c nude mice were purchased from the animal house of Shanghai University of Traditional Chinese Medicine. All animal experiments followed the ethics of animal experimentation, and the animal experiments were approved by the Animal Ethics Committee of Shanghai University of Traditional Chinese Medicine (No. PZSHUTCM2401170001). All animals were housed in the SFP‐class animal house of the Animal Testing Center of Shanghai University of Traditional Chinese Medicine. The temperature was maintained at 22±2 °C and relative humidity at 50±10% according to the 12‐h rhythm of day and night each.

The hrHL cells were provided by Prof. Pan Guoyu from the Chinese Academy of Sciences, and cultured according to the instructions provided by the laboratory. HUVEC cells were purchased from Zhongqiao Xinzhou Company (No.PRI‐H‐00023), and cultured in ECM complete medium. HUMSC (Zhongqiao Xinzhou Co. DF‐GMP‐ZB09BA) were provided by Prof. Yu Tingting's group from Nanjing Medical University, and cultured in F12 complete medium. LX‐2 cells were purchased from Zhongqiao Xinzhou Company (No. ZM0026, CVCL_5792)), and cultured in DMEM complete medium. THP‐1 cells (Zhongqiao Xinzhou Co. No.ZM0086, CVCL_0006) were obtained from the Institute of Cross‐Science and cultured in 1640 complete medium. The above cells were cultured in an incubator at 37 °C with 5% CO_2_.

### Formation of hrHL Only and Co‐Cultured Organoids

rHLOs were formed by following the protocol: after the hrHL cells were matured and counted, the plates were spread according to the number of every 3000 cells per well, and the specific U‐bottom low‐adhesion 96‐well plate (Corning: No.7007) was selected. The rHLOs were formed after 5 days of culture.

3HLOs construction: hrHL cells, HUVECs, and HUMSCs were mixed at a ratio of 10:5:2, then cultured in 3HLO complete medium for 5 days using specialized U‐bottom ultra‐low attachment 96‐well plates (Corning, No. 7007). The organoids formed after 5 days of culture.

hSMOs construction: hrHL cells, LX‐2 cells, and THP‐1 cells were mixed at a ratio of 10:2:1 and cultured in hSMOs complete medium for 5 days using specific U‐bottom low‐attachment 96‐well plates (Corning, No. 7007). Organoids formed after 5 days of culture. Subsequently photographed by live cell holotomography microscopy (tomocube HT‐X1). Fluorescent dyes were used to distinguish cell types by pre‐labeling cells before co‐culturing. The mitochondrial red dye (Beyotime Co. No.C1032) was selected and stained for 15 min at a ratio of 1:1000. DAPI dye was used for nuclear staining and labeling and stained for 30 min at a ratio of 1:2000. (Beyotime Co. No.C1042M), diluted in the ratio of 1:2000, staining for 15 min is sufficient. Observation by the ECHO optical instrument.^[^ ^61^, ^62^ ^]^ Liver organoid microvessel microchips were fabricated by molding PDMS to form the outer shell, punching holes to connect to the channels and reservoirs using 3 mm and 5 mm punches, and adhering PDMS to the slides by plasma treatment. Superelastic nickel needles with a diameter of 220 µm and a length of 3 cm were fixed in the center of the cavity and secured with SYLGARD 160A and SYLGARD 160B. They were sterilized by alcohol and UV sterilization before use.

### Construction of Liver Organoid Microvascular Chip

Mouse tail type I collagen (Corning, 354249) was neutralized and diluted to 7 mg ml^−1^ with DI water, 1N sodium hydroxide (S2770, Sigma), 10 × DMEM. After neutralization, the organoids were embedded in collagen solution to give a final concentration of 5 spheres µl^−1^, which were injected in the middle of two small wells so that they were randomly distributed in the cavity. The collagen was then gelatinized at 37 °C for 15 min, and agarose was heated and injected at the ends of the wells to support the collagen and prevent collapse. A small amount of organoids medium was added to the reservoir to prevent drying out and stored in a refrigerator at 4 °C for 6 h to remove air bubbles, followed by removal of the needle to form a cylindrical channel in the gel and perfusion with organoids medium for 1 day. GFP‐HUVEC was introduced into the channel at a concentration of 107 /ml and allowed to adhere to the channel wall for 2 h. After that, the device was incubated under a constant flow (1 ml h^−1^).

### PAS Staining

PAS staining, that is, the periodic acid Schiff method, was a method of glycogen staining, which can be used to demonstrate and identify the nature of intracellular vacuolated, organoid tissue in paraffin sections 7um. First, the paraffin sections were placed in an oven at 60 °C for 1–2 h; paraffin sections were routinely de‐waxed to water with xylene, ethanol, and then oxidized in 0.5% aqueous periodic acid for 5 min, rinsed in distilled water for more than 5 min, and Schiff's Reagent Action 10–30 min. Subsequently, aqueous sulfite was washed three times for 1.5–2 min each time, running water for 15 min, Harry's hematoxylin stained the nuclei lightly for 1–2 min (too dark to differentiate), followed by running water rinsing, and, finally, dehydrated, transparent, and sealed as usual.

### ICG and Rho‐123 Uptake and Efflux Experiments

1 mg mL^−1^ indocyanine green (ICG) (Daiichi Sankyo, Tokyo, Japan) was selected as the reagent to be used, and it was diluted according to the instructions of the working concentration to be used, and then co‐cultivated with the organoids in an incubator at 37 °C for 30 min, and then the organoids' uptake of ICG was observed and recorded by using an ECHO light microscope. ICG uptake. At the end of the observation, the medium mixed with ICG was discarded, a new complete medium was added, and the excretion of ICG was observed after 6 h and recorded by ECHO optical microscope.

For Rhodamine 123 (Rho‐123) uptake and efflux experiments, Rho‐123 (original leaf Co., No. S19123) was selected to be prepared according to the instructions, and then formulated with complete culture medium for organoids to a working concentration of 5 µm, and placed in an incubator at 37 °C for 30 min, and then the uptake of Rho‐123 in organoids was observed and recorded by using an ECHO optical microscope. The uptake of Rho‐123 by the organoids was observed and recorded using an ECHO optical microscope, and the fluorescence module, FITC, was selected. At the end of the observation, the medium mixed with Rho‐123 was discarded, fresh complete medium was added, and the expulsion of Rho‐123 was observed and recorded by an ECHO optical microscope after 6 h. The uptake of Rho‐123 in the organoids was recorded using an ECHO optical microscope.

### Vascular Permeability Assay

First, prepare 70 kDa dextran (MCE Co., FITC, No.60842‐46‐8). It was prepared to a working concentration of 100 mM as required for the experiment. The chip was removed from both ends of the pool and pipetted to discard the liquid in the reservoirs at both ends, and then 100 µl of medium containing 70 kDa dextran was added at one end, while 10 µl the other at the other end without fluorescence dye. Subsequently, it was transferred to an ECHO fluorescence microscope for observation, and the penetration of the 70 kDa dextran was recorded for 10–15 min, and photographs were taken at intervals of 30 s. Phase contrast and fluorescence images (8107 µm × 664 µm) were acquired every 30 s for 1 min before and 5 min following perfusion with the fluorescent solutes. Permeability of microvessels was calculated from P = (r/2)(1/∆I)(dI/dt), where r is the vessel radius, ∆*I* is the increase in fluorescence intensity upon initiation of perfusion of the solute, and dI/dt is the rate of increase of fluorescence as the solute permeates into the collagen gel.

### Subcutaneous Loading of Liver Organoids in Nude Mice

Liver organoids were first cultured, and the state of the organoids was observed under a microscope and counted, with 3000‐5000 organoids loaded subcutaneously in each nude mouse. After counting, the organoids were centrifuged and mixed with 200 µl (for one nude mouse) of Matrigel gel (Corning, No. 356 231) and placed on ice for use. Then the nude mice were taken out, and their backs were sterilized, and a 1 ml syringe was selected for subcutaneous injection, taking care not to inject too deeply, and the depth of the injection should be enough to stay under the skin. Subsequently, the growth size of the organoid was observed and recorded every day, calculated by the formula: long diameter^*^short diameter^2^/2.

### Construction of an Animal Model of Alcoholic Liver Injury

The NIAAA alcoholic liver disease model was constructed. The modeling method of NIAAA alcoholic liver disease was usually divided into the transition period, the period of feeding alcoholic liquid feed, and the period of high alcohol gavage modeling. After the transition period, the alcoholic liquid feed was continuously fed for 10 days, and then the alcoholic liquid feed was continuously gavaged for three days with a high level of white wine (alcohol content of 52 degrees or above), at a dosage of 5 g kg^−1^ of body mass. Mass, blood was collected, and relevant indicators were tested. Lieber‐DeCarli (LD No. 1258&1259) liquid feed was used, the initial state of the feed was an oil‐containing powdered feed, according to different ways of configuration, two kinds of liquid feed can be configured, that is, alcohol‐containing alcohol liquid feed group and non‐alcohol‐containing control feed, configuring the alcohol liquid feed, the feed powder was 132.18 g mixed with 817.82 g of water, and then added alcohol 50 g, mixed well again, and the remaining unused, sealed and refrigerated. For the control group, 132.18 g of feed powder, add 89.6 g of maltodextrin, add 778.22 g of water, and mix well. Regarding the feeding of alcohol liquid feed: alcohol liquid feed, because the configuration was a paste‐like fluid state, the conventional water bottle was easy to be blocked, so it is recommended to purchase a water bottle with a ball spout or a special feeding bottle for liquid feed.

Based on the success of the animal model of alcoholic liver disease, the drug group of fenofibrate, apigenin, & thaliotrine was chosen, where the dose of fenofibrate was 100 mg kg^−1^, the apigenin dose was 50 mg kg^−1^, and the thaliotrine dose was 10 mg kg^−1^. The drugs were administered by intraperitoneal injection.

### Statistical Analysis

The data obtained in the experiment were analyzed: statistical analysis was performed using Graphpad Prism 9.0.0. Students' *t*‐test was used to analyze the difference between two independent samples; one‐way ANOVA was used to analyze the comparison between multiple groups. The SNK‐q test was used for multiple comparisons; Pearson correlation was used to analyze the correlation between variables in two groups.

## Conflict of Interest

The authors declare no conflict of interest.

## Supporting information



Supporting Information

Supplemental Movie 1

Supplemental Movie 2

Supplemental Movie 3

Supplemental Movie 4

Supplemental Movie 5

## Data Availability

The data that support the findings of this study are available from the corresponding author upon reasonable request.
