# Supplementary material for: Novel Vascularized Human Liver Organoids for Modeling Alcohol‐Induced Liver Injury and Developing Hepatoprotective Therapy
Source: Adv Sci (Weinh). 2025 Dec 5;13(9):e11169. doi: 10.1002/advs.202511169 (PMC12904048; doi:10.1002/advs.202511169)
Supplement: Supplementary file 1 — Supporting Information [file ADVS-13-e11169-s002.docx]

**Supplemental Information**

**Novel Vascularized Human Liver Organoids for Modeling Alcohol-induced Liver Injury and Developing Hepatoprotective Therapy**

*Kangdi Yang, Xiayan Chu, Xuerui Wang, Wenkun Zhang, Jinnuo Lu, Chuting Xu, Shoucheng Hu, Guoyu Pan, Chih-Tsung Yang, Xiaohui Zhang, Shaojin Li, Zhaobin Guo *, Hanyang Liu*, and Guangbo Ge**

* Corresponding author. State Key Laboratory of Discovery and Utilization of Functional Components in Traditional Chinese Medicine; Shanghai Frontiers Science Center of TCM Chemical Biology; Institute of Interdisciplinary Integrative Medicine Research, Shanghai University of Traditional Chinese Medicine, Shanghai, 201203 China

E-mail addresses:

[guozhaobin@shutcm.edu.cn](mailto:guozhaobin@shutcm.edu.cn) (Z. Guo)

[brandenliu@live.com](mailto:brandenliu@live.com) (H.Liu)

[geguangbo@shutcm.edu.cn](mailto:geguangbo@shutcm.edu.cn) (G. Ge)

**This file contains supplementary experimental details, 7 supplementary figures and 2 supplementary tables.**

**Contents**

Supplementary experimental details and methods 3

Figure S1 13

Figure S2 17

Figure S3 18

Figure S4 20

Figure S5 22

Figure S6 24

Figure S7 25

Table S1 26

Table S2 26

Reference27

**Supplementary experimental details and methods**

**HE staining**

Paraffin sections were examined and baked at 62°C for 1 h. Sections were deparaffinized: tissue chips were placed in xylene for 20 min, replaced with xylene for another 20 min; hydration: anhydrous ethanol for 1 min; anhydrous ethanol for 1 min; 95% ethanol for 1 min; 70% ethanol for 1 min; Wash in tap water for 5 min; wash in distilled water; add one drop or 100 μl of hydrogen peroxide blocking solution to each slice and incubate for 10 min at room temperature to block endogenous peroxidase activity. Thermal antigen repair (1 mmol Tris-EDTA PH=9.0): the washed slices were placed on top of plastic racks in boiling antigen repair solution, and the boiling was maintained for After 15min, keep warm for 15min, turn off the power and cool down naturally. Wash with PBS 3 times, 5 min each time; remove PBS, add 100 µl of 5% BSA blocking solution to each slice, incubate at room temperature for 20min.Remove serum, add 100 µl of the first primary antibody dilution of primary antibody to each slice, and react at 4℃ overnight; the next day remove the humidified box, and leave it in room temperature for 1h or 37℃ for 30min; the next day remove the humidified box, and leave it in room temperature for 1h or 37℃ for 30min. The next day, remove the wet box and leave it at room temperature for 1h or 37℃ for 30min; wash with PBS 3 times, 5 min each time; remove the PBS and add 100µl of labeled secondary antibody to each slice, incubate at 37℃ for 30 min; wash with PBS 3 times, 5min each time; remove the PBS and add 100 µl of try-488 tyramine converting reagent to each slice, incubate at room temperature for 10-30 min; wash with PBS 3 times, 5min each time; repeat the operation Repeat steps 6-13; add 100µl of the second diluted primary antibody at step 9; remove PBS, add 100µl of try-cy3 tyramine-converting reagent to each slice, incubate at room temperature for 10-30min; wash with PBS 3 times, 5min each time; remove PBS, add 100µl of fluorescence-quenching sealer (containing DAPI) to each slice. (containing DAPI). Subsequently, the slices were photographed by Leica SP8 confocal microscope and quantitatively analyzed as well as reconstructed by ImageJ.

**IF staining and analysis**

Paraffin sections were examined and baked at 62°C for 1 h. Sections were deparaffinized: tissue chips were placed in xylene for 20 min, replaced with xylene for another 20 min, replaced with xylene for another 20 min; hydration: anhydrous ethanol for 1 min; anhydrous ethanol for 1 min; 95% ethanol for 1 min; 70% ethanol for 1 min; Wash in tap water for 5 min; wash in distilled water; add one drop or 100 µl of hydrogen peroxide blocking solution to each slice and incubate for 10 min at room temperature to block endogenous peroxidase activity. Thermal antigen repair (1 mmol Tris-EDTA PH=9.0): the washed slices were placed on top of plastic racks in boiling antigen repair solution, and the boiling was maintained for After 15min, keep warm for 15min, turn off the power and cool down naturally. wash with PBS 3 times, 5min each time; remove PBS, add 100µl of 5% BSA blocking solution to each slice, incubate at room temperature for 20min. remove serum, add 100µl of the first primary antibody dilution of primary antibody to each slice, and react at 4℃ overnight; the next day remove the humidified box, and leave it in room temperature for 1h or 37℃ for 30min; wash with PBS 3 times, 5 min each time; remove the PBS and add 100µl of labeled secondary antibody to each slice, incubate at 37℃ for 30min; wash with PBS 3 times, 5min each time; remove the PBS and add 100µl of try-488 tyramine converting reagent to each slice, incubate at room temperature for 10-30min; wash with PBS 3 times, 5min each time; repeat the operation Repeat steps 6-13; add 100µl of the second diluted primary antibody at step 9; remove PBS, add 100µl of try-cy3 tyramine-converting reagent to each slice, incubate at room temperature for 10-30min; wash with PBS 3 times, 5min each time; remove PBS, add 100µl of fluorescence-quenching sealer (containing DAPI) to each slice. (containing DAPI). Subsequently, the slices were photographed by Leica SP8 confocal microscope and quantitatively analyzed as well as reconstructed by ImageJ.

**IHC staining and analysis**

Paraffin sections were baked at 62°C for 1 h. Sections were dewaxed: tissue chips were soaked in xylene for 20 min, replaced with xylene for another 20 min, replaced with xylene for another 20 min; hydration: 1 min in anhydrous ethanol; 1 min in anhydrous ethanol; 1 min in 95% ethanol; 1 min in 95% ethanol; 1 min in 70% ethanol; Wash in tap water for 5 min; Wash in distilled water; Add one drop or 100µl of hydrogen peroxide blocking solution to each slice and incubate at room temperature for 10 min to block endogenous peroxidase activity. Wash in PBS 3 times for 5 min each time; Thermal Antigen Repair (1mmol Tris-EDTA PH=9.0): place washed slices onto the top of a plastic rack in a boiling Thermal antigen repair (1mmol Tris-EDTA PH=9.0): put the washed slices into the plastic rack, put them into the boiling antigen repair solution, keep boiling for 15min, keep warm for 15min, turn off the power and cool naturally, wash with PBS for 3 times, each time for 5min; remove the PBS, add 1 drop or 100µl of 5% BSA blocking solution to each slice, and incubate for 20min at room temperature, remove the serum, add 1 drop or 50µl of primary antibody dilution to each slice, and react at 4℃ overnight; The next day, remove the humidor and leave it at room temperature for 1h or at 37℃ for 30min; wash with PBS 3 times, 5min each time; remove PBS and add 1 drop or 50µl of labeled secondary antibody to each slice, incubate at 37℃ for 30min; wash with PBS 3 times, 5min each time; remove PBS, and add DAB to each slice to develop the color, and grasp the extent of the staining under the microscope (when yellow granules or flaky precipitates appeared, the color of the staining solution would not be changed). When there are yellow particles or flaky precipitates, terminate the staining immediately); rinse clean the DAB residual solution with tap water, re-stain with hematoxylin for 30s, rinse with tap water for 5min. hydrochloric acid alcohol differentiation for 1s, rinse with tap water to return to the blue for 10min; dewatering: soak in 70% ethanol for 3min; 95% ethanol for 3min; 95% ethanol for 3min; anhydrous ethanol for 3min for two times; transparency: the tissues are placed in xylene for 5 minutes three times; sealing: neutral gum sealing with coverslips. Sections were then scanned using a KFBIO KF-PRO-120 digital pathology slide scanner. Positive rates were analyzed by ImageJ.

**Constructing an alcoholic liver disease model using organoids**

After the organoids were cultured and matured, alcohol was added to the medium at a concentration of 100 mM, and then the medium was co-cultivated with the organoids for 72 h. During this period, we chose to seal the petri dish with a sealing film in order to prevent the decrease of alcohol concentration caused by the volatilization of alcohol. Since the microfluidic chip could not be completely sealed, we chose to change the liquid treatment every 12 hours to maintain the alcohol concentration.

Detection of alcoholic liver disease model, ROS detection: we chose the ROS kit (Beyotime Co., No. S0033M) for detection, and diluted DCFH-DA with serum-free culture solution according to 1:1000 to make the final concentration of 10μmol/liter. The organoids were collected and suspended in diluted DCFH-DA and incubated for 20 min at 37°C in a cell culture incubator. The mixing was inverted every 3-5 minutes to make full contact between the probe and the organoids. Cells were washed three times with serum-free cell culture medium to fully remove DCFH-DA that did not enter the organoid. the cells were subsequently observed and recorded using an ECHO light microscope and analyzed for brightness by ImageJ. JC-10 assay: the JC-10 kit was selected for the assay (Beyotime Co., No. C2003S). The amount of JC-10 staining working solution required for each group was 1 ml, and the amount of JC-10 staining working solution for other culture vessels was followed by analogy. Take appropriate amount of JC-10 (200X) and dilute JC-1 at the ratio of 1 ml of JC-10 staining buffer for every 5µl of JC-1 0 (200×). use a pipette to blow repeatedly and mix well to make JC-10 staining working solution. Take the organoid and resuspend it in 0.5 ml of cell culture medium, which can contain serum and phenol red. Add 0.5 ml of JC-10 staining working solution, invert several times and mix well. Incubate the cells in a cell incubator at 37°C for 20 min. At the end of the incubation at 37°C, centrifuge the cells at 600g for 3-4 min at 4°C to precipitate the cells. Discard the supernatant, taking care to try not to aspirate the organoid. Wash twice with JC-10 staining buffer: add 1 ml of JC-10 staining buffer to resuspend the organoids, centrifuge at 600g for 3-4 minutes at 4℃, precipitate the organoids and discard the supernatant. Add 1ml of JC-10 staining buffer to resuspend the organoid, centrifuge at 600g for 3-4 minutes at 4℃, precipitate the organoid and discard the supernatant. After resuspending with appropriate amount of JC-10 staining buffer again, the organs were observed by fluorescence microscope or laser confocal microscope, and also detected by fluorescence spectrophotometer or analyzed by flow cytometry. Finally, quantitative analysis was performed by ImageJ. Live-dead staining assay: live-dead dye kit was selected for the assay (Beyotime Co., No. C2015L). The organoids were washed the cells with PBS for 1 time; centrifuged at 250-1000×g for 5 min at room temperature, the supernatant was aspirated, and washed with PBS for 1 time. Phenol red or serum is interfering with the detection of this kit, it is better to use a vacuum pump when aspirating the culture fluid and PBS. In the case that the residual liquid can be adequately aspirated, it can be washed without PBS. Staining. Add the appropriate volume of Calcein AM/PI assay working solution. incubate for 30 min at 37°C away from light. detection. At the end of incubation, observe the staining effect under a fluorescence microscope (Calcein AM is green fluorescence, Ex/Em=494/517nm; PI is red fluorescence, Ex/Em=535/617nm). Further staining with other fluorescence was performed, such as staining the nucleus with Hoechst 33342 Live Cell Staining Solution (C1027/C1028/C1029). Note that the whole process should be protected from light. Laica SP8 was photographed and quantitatively analyzed by ImageJ.

Based on the success of the alcoholic liver disease model, we chose fenofibrate (Sigam No. F6020 CAS49562-28-9) as the positive drug, and apigenin (Sigma No. 42251 CAS.520-36-5) with thaliotrine (CAS. 2141-09-5) as the test drug group, where the fenofibrate dose was 100 μM, apigenin dose was 2.5μM and thaliotrine dose was 50μM.

**Construction of an animal model of alcoholic liver ingury**

Our NIAAA alcoholic liver disease model was constructed. The modeling method of NIAAA alcoholic liver disease is usually divided into the transition period, the period of feeding alcoholic liquid feed, and the period of high alcohol gavage modeling. After the transition period, the alcoholic liquid feed was continuously fed for 10 days, and then the alcoholic liquid feed was continuously gavaged for three days with a high level of white wine (alcohol content of 52 degrees or above), at a dosage of 5 g/kg of body mass. Mass, blood was collected, and relevant indicators were tested. We used Lieber-DeCarli (LD No. 1258&1259) liquid feed, the initial state of the feed is an oil-containing powdered feed, according to different ways of configuration, two kinds of liquid feed can be configured, i.e., alcohol-containing alcohol liquid feed group and non-alcohol-containing control feed, configuring the alcohol liquid feed, the feed powder was 132.18 g mixed with 817.82 g of water, and then added alcohol 50g, mixed well again, and the remaining unused, sealed and refrigerated. For the control group, 132.18g of feed powder, add 89.6g of maltodextrin, add 778.22g of water and mix well. Regarding the feeding of alcohol liquid feed: alcohol liquid feed, because the configuration is a paste-like fluid state, the conventional water bottle is easy to be blocked, so it is recommended to purchase a water bottle with a ball spout or a special feeding bottle for liquid feed.

Based on the success of the animal model of alcoholic liver disease, we chose the drug group of fenofibrate, apigenin, & thaliotrine, where the dose of fenofibrate was 100 mg/Kg, apigenin dose was 50 mg/Kg, and thaliotrine dose was 10 mg/Kg. the drugs were administered by intraperitoneal injection.

**Physiological and biochemical indicators testing**

Immediately after blood collection, place the blood into a 1.5 mL centrifuge tube (without anticoagulant or preservative). Allow the blood to stand at room temperature for 30 - 60 minutes. Centrifuge the blood sample at a relative centrifugal force (RCF) of 14,000 g for 10 - 15 minutes, separate the serum by pipetting, and transfer to a clean centrifuge tube. The serum sample may be centrifuged at 14,000 g for an additional 2 - 3 minutes to separate any remaining red blood cells. The separated serum is transferred to a clean centrifuge tube. The indicators in the serum are then measured using a fully automated biochemical analyzer based on the principle of photoelectric colorimetry mainly for liver and kidney function, blood glucose, and blood lipids.

Test content: **a** Liver function: (total protein (TP), albumin (ALB), total bilirubin (TBIL), alanine aminotransferase (ALT), albumin oxalate aminotransferase (AST), alkaline phosphatase (ALP), cholinesterase (CHE), glutamyl transpeptidase (GGT), direct bilirubin (DBIL);

**b** Renal function: creatinine (CRea), urea (UREA), uric acid (UA), urinary protein (UP);

**c** Lipids: triglycerides (TG), cholesterol (T-CHO), low-density lipoprotein cholesterol (LDL-C), high-density lipoprotein cholesterol (HDL-C);

**d** Cardiac enzymes: lactate dehydrogenase (LDH), creatine kinase (CK);

**e** Other testing items: blood glucose GLU, etc., of which ALT, AST in cell supernatants are also detected using this instrument.

**mRNA sequencing analysis process**

RNA Sequencing and Differentially Expressed Genes Analysis The libraries were sequenced on a lumina Novaseq 6000 platform and 150 bp paired-end reads were generated. Raw reads of fast format were firstly processed using fast and the low-quality reads were removed to obtain the clean reads. The clean reads were mapped to the reference genome using HISAT2. FPKM of each gene was calculated and the read counts of each gene were obtained by HT Seq-count. PCA analysis were performed using R (v 3.2.0) to evaluate the biological duplication of samples. Differential expression analysis was performed using the DESeq2. Q value < 0.05 and foldchange > 2 or foldchange < 0.5 was set as the threshold for significantly differential expression gene (DEGs). Hierarchical cluster analysis of DEGs was performed using R (v 3.2.0) to demonstrate the expression pattern of genes in different groups and samples. The radar map of top 30 genes was drawn to show the expression of up-regulated or down-regulated DEGs using R packet grader. Based on the hypergeometric distribution, GO, KEGG pathway, Reactome and WikiPathways enrichment analysis of DEGs were performed to screen the significant enriched term using R (v 3.2.0), respectively. R (v 3.2.0) was used to draw the column diagram, the chord diagram and bubble diagram of the significant enrichment term. Gene Set Enrichment Analysis (GSEA) was performed using GSEA software. The analysis waswww.oebiotech.com used a predefined gene set, and the genes were ranked according to the degree of differential expression in the two types of samples. Then it is tested whether the predefined gene set was enriched at the top or bottom of the ranking list.

**Sample Preparation**

Samples stored at -80 ℃ were thawed at room temperature. 500μL of sample was added to a 1.5 mL Eppendorf tube with 500μL of L-2-chlorophenylalanine (0.06 mg/mL) dissolved in methanol as internal standard, and the tube was vortexed for 10 s. Subsequently, 500μL of ice-cold mixture of methanol and acetonitrile (2/1, vol/vol) was added, and the mixtures were vortexed for 1 min, and the whole samples were extracted by ultrasonic for 10 min in ice-water bath，stored at -20 ℃ for 30 min. The extract was centrifuged at 4°C (13000 rpm) for 10 min. 500 μL of supernatant in a glass vial was dried in a freeze concentration centrifugal dryer .500μL mixture of methanol and water (1/4, vol/vol) were added to each sample, samples vortexed for 30 s, extracted by ultrasonic for 3 min in ice-water bat, then placed at -20°C for 2 h. Samples were centrifuged at 4°C (13000 rpm) for 10 min. The supernatants (500 μL) from each tube were collected using crystal syringes, filtered through 0.22μm microfilters and transferred to LC vials. The vials were stored at -80°C until LC -MS analysis. QC samples were prepared by mixing aliquot of the all samples to be a pooled sample.

**Metabolomic profiling by LC-MS/MS**

The metabolomic data analysis was performed by Shanghai Luming biological technology co., LTD (Shanghai, China). An ACQUITY UPLC I-Class plus (Waters Corporation, Milford, USA) fitted with Q-Exactive mass spectrometer equipped with heated electrospray ionization (ESI) source (Thermo Fisher Scientific, Waltham, MA, USA) was used to analyze the metabolic profiling in both ESI positive and ESI negative ion modes. An ACQUITY UPLC HSS T3 column (1.8μm, 2.1 × 100 mm) were employed in both positive and negative modes. The binary gradient elution system consisted of (A) water (containing 0.1 % formic acid, v/v) and (B) acetonitrile (containing 0.1 % formic acid, v/v) and separation was achieved using the following gradient: 0.01 min, 5% B; 2min, 5% B; 4min, 30% B; 8min, 50% B; 10min, 80% B; 14min, 100% B; 15 min, 100% B; 15.1 min, 5% and 16 min, 5%B. The flow rate was 0.35 mL/min and the column temperature was maintained at 45℃. All the samples were kept at 10℃ during the analysis. The injection volume was 500 μL. The mass range was recorded from *m/z* 100 to 1,000. The resolution was set at 70,000 for the full MS scans and 17500 for HCD MS/MS scans. The Collision energy was set at 10, 20 and 40 eV. The mass spectrometer operated as follows: spray voltage, 3800 V (+) and 3200 V (−); sheath gas flow rate, 35 arbitrary units; auxiliary gas flow rate, 8 arbitrary units; capillary temperature, 320°C; Aux gas heater temperature, 350°C; S-lens RF level, 50.

**Data Preprocessing and Statistical Analysis**

The original LC-MS data were processed by software Progenesis QI V2.3 (Nonlinear, Dynamics, Newcastle, UK) for baseline filtering, peak identification, integral, retention time correction, peak alignment, and normalization. Main parameters of 5 ppm precursor tolerance, 10 ppm product tolerance, and 5% product ion threshold were applied. Compound identification was based on precise mass-to-charge ratio (M/z), secondary fragments, and isotopic distribution using The Human Metabolome Database (HMDB), Lipid maps (V2.3), Met lin, and self-built databases. The extracted data were then further processed by removing any peaks with a missing value (ion intensity = 0) in more than 50% in groups, by replacing zero value by half of the minimum value, and by screening according to the qualitative results of the compound. Compounds with resulting scores below 36 (out of 60) points were also deemed to be inaccurate and removed. A data matrix was combined from the positive and negative ion data.

The matrix was imported in R to carry out Principal Component Analysis (PCA) to observe the overall distribution among the samples and the stability of the whole analysis process. Orthogonal Partial Least-Squares-Discriminant Analysis (OPLS-DA) and Partial Least-Squares-Discriminant Analysis (PLS-DA) were utilized to distinguish the metabolites that differ between groups. To prevent overfitting, 7-fold cross-validation and 200 Response Permutation Testing (RPT) were used to evaluate the quality of the model. Variable Importance of Projection (VIP) values obtained from the OPLS-DA model were used to rank the overall contribution of each variable to group discrimination. A two-tailed Student’s T-test was further used to verify whether the metabolites of difference between groups were significant. Differential metabolites were selected with VIP values greater than 1.0 and p-values less than 0.05. Differential metabolites were further used to for KEGG pathway (http://www.genome.jp/kegg/) enrichment analysis.

**Proteolytic from Organoid-Chip digestion (Culture Supernatant)**

Protein samples (10 μg) were loaded into a 10 kDa ultrafiltration tubes and centrifuged at 12,000 × g for 20 min. Subsequently, 200 μL of 50 mM ammonium bicarbonate was added for buffer exchange. The sample was reduced with 200 μL of 5 mM dithiothreitol (DTT) at 56 °C for 30 min, followed by centrifugation to remove the reaction solution. Alkylation was performed by adding 200 μL of 11 mM iodoacetamide (IAA) and incubating in the dark at room temperature for 20 min. After centrifugation, the reaction solution was discarded. Trypsin (0.25 μg, dissolved in 40 μL of 50 mM ammonium bicarbonate) and 80 μL of 50 mM ammonium bicarbonate were added to the filter, and then the mixture was incubated at 37°C overnight. The enzymatic reaction was terminated by adding 20 μL of 10% formic acid, and the filtrate was collected via centrifugation (12,000 × g). To ensure complete peptide recovery, 200 μL of 50 mM ammonium bicarbonate was added to the filter, and the centrifugation step was repeated twice. All filtrates were pooled and vacuum-concentrated until completely dry. The dried peptides were reconstituted in 20 μL of 0.1% formic acid, sonicated for 5 min, and centrifuged at 12,000 rpm for 20 min. Finally, 15 μL of the supernatant was transferred into an injection vial for nanoLC-MS/MS analysis.

**Proteome profiling of the plasma from mice**

The EasyPep DeeP Low-Abundance Protein Enrichment and Pre-treatment Kit (Cat. No. OSFP0002) was used to capture low-abundance proteins in plasma samples using functional group-modified superparamagnetic beads. This technology relies on non-covalent interactions (e.g., electrostatic forces) between surface ligands on the functionalized beads and target proteins, enabling rapid and efficient enrichment of low-abundance proteins through magnetic separation. The workflow involved the following steps: The sample was mixed with the beads and incubated at 37°C for 1 h. After magnetic bead collection, unbound proteins were removed by washing. Reagent A was added to lyse and reduce/alkylate proteins under 95°C incubation for 5 min. Subsequently, Reagent B was introduced for enzymatic digestion at 37°C for 2 h. The reaction was terminated by adding Reagent C, followed by centrifugation at 20,000 × g for 1 min. The supernatant was collected for desalting prior to proteome profiling.

**Analytical conditions of nanoLC-MS/MS analysis**

Samples were analyzed using a Thermo Vanquish Neo UHPLC system coupled with a Thermo Scientific™ Orbitrap™ Astral™ mass spectrometer, equipped with an ES906 analytical column. A 23-min gradient method was applied as follows: Phase B (80% acetonitrile/20% water) increased from 4.0% to 8.0% over 6 s at a flow rate of 1.3 μL/min, followed by a gradient from 8.0% to 35.0% Phase B over 20.2 min at 0.8 μL/min. Subsequently, Phase B rose from 35.0% to 55.0% in 24 s (0.8 μL/min), then to 99.0% in 30 s (2.0 μL/min), and was then maintained at 99.0% for 1 min (2.0 μL/min). The instrument operated in positive ion mode with a charge state of 2, a maximum injection time of 5 ms, and a scan range of m/z 380–980 at a resolution of 240,000 for MS2 spectra acquisition. Data-Independent Acquisition (DIA) mode was employed with an isolation window width of 2 m/z, normalized collision energy of 25%, and a scan range of *m/z* 150–2000.

**Data Processing**

Raw data were processed using Spectronaut 19.4, with protein database searches performed against the human and mouse proteome databases retrieved from UniProt.org. Carbamidomethylation was set as a fixed modification, while oxidation (M) and protein N-terminal acetylation were assigned as variable modifications, allowing a maximum of five variable modifications per peptide. Trypsin was selected as the protease with a maximum of two missed cleavages permitted. Peptide length was restricted to 7-52 amino acids. Quantitative analysis was performed using MS2 data with peak area-based quantification. Default parameters were applied for all other settings.

**Real time qPCR**

Firstly, total RNA extraction was performed, cells were removed and RNA was extracted with RNA extraction kit (Feijie, 220011), RNA concentration was detected to ensure the quality of extraction. After that, RNA was reverse transcribed to cDNA. reverse transcription PCR system (20 μl):

| **Components** | **Volume** |
| --- | --- |
| 5× Buffer | 4μl |
| 4× DNTP | 1μl |
| Oligdt | 1μl |
| Reverse transcriptase | 0.25μl |

Add RNA 500ng and DEPC water to 20μl, reverse transcription was performed at 42℃ for 59 min and 72℃ for 15 min. The reverse transcribed cDNA was collected for quantitative PCR. then RT-qPCR was performed to quantify the reverse transcribed cDNA with quantitative primers (**Table S2.**).

Quantification system (20 μl):

| **Components** | **Volume** |
| --- | --- |
| Upstream primer 10μm | 1μl |
| Downstream primer 10μm | 1μl |
| ddH_2_O | 7μl |
| cDNA template | 2μl |
| Master Mix enzyme | 10μl |

Quantification was accomplished on an ABI7500 machine with 40 cycles of 95°C for 10s, 58°C for 15s, and 72°C for 35s.

Data analysis was performed, and Ct values for each sample, according to 2^-ΔΔCt^ to calculate the relative mRNA expression.

**S1. Organoid Comprehensively Boosted the Hepatic Functions of hrHL**

HrHL were developed by inserting genes of various liver transcription factors including FOXA3, HNF1, and HNF4A into fibroblasts. These modifications endowed the cells with hepatocyte functions and *in vitro* expanding capability. RHLOs was created by seeding hrHL onto U-shaped, ultralow adhesion plates (**Figure S1A**), following the protocol described by Hui. et. al.^[1],[2]^ Cells formed loose aggregates at 6 hours and compacted at 24 hours, indicating robust cell-cell interactions within the first 24h (**Figure S1B-C**). Subsequently, the organoids expanded significantly starting from day 2, suggesting that hrHL within the organoids were proliferating. Immunofluorescence staining (IF) confirmed these findings, showing significant expression of the cell-cell adhesion marker E-Cadherin and the proliferation marker Ki67(**Figure S1D**). Additionally, liver bile duct markers, such as epithelial cell adhesion molecule (EpCAM) and cytokeratin 19 (CK-19), were strongly expressed (**Figure S1D**). Lumen structures were observed in hematoxylin-eosin (HE) staining (**Figure S1E**), suggesting preliminary bile duct formation. Although the profound expression of albumin (ALB) indicated strong biosynthetic functions, the substantial expression of fetal liver marker alpha-fetoprotein (AFP) still raises our concern regarding the maturity of the rHLOs (**Figure S1D**).

To assay the hepatic functions of the rHLOs, several tests were performed, including glycogen storage and transport. Significant PAS (Periodic Acid-Schiff) positive staining was observed within the organoids (**Figure S1F**), along with high levels of intake and excretion of both indocyanine green (ICG) and Rhodamine (Rho-123) (**Figure S1G**). Notably, ICG uptake was concentrated at the edges of the rHLOs, while Rho-123 was taken up throughout the organoids. This could due to the natural (e.g. molecular weight) of different dyes, or the different imaging modalities (optical vs fluorescence). In addition, holotomographic microscopy (HTM) was used to monitor the structural integrity of the organoid. Using cellular holotomography, we were able to clearly visualize the microstructure of the cells, including tight junctions, internal vacuolization, and organelles such as the cell nucleus (red square), mitochondria (orange square), lipid droplets (yellow square), lysosomes (green square), and bile canaliculus-like structures (blue square) (**Figure S1H**). Multiple omics analyses were conducted to compare hrHL grown in traditional 2D culture to those grown in organoids. PCA of RNA-seq data revealed substantial differences between the 2D and organoid conditions (**Figure S1I**). KEGG analysis showed significant enrichment of genes related to hepatic functions, including ANKRD1, KRT4, CYP2C9, and COL21A1, in organoids. GO analysis demonstrated enhanced endogenous and exogenous metabolism in organoids compared to 2D cultures, with enriched biological processes such as cholesterol homeostasis, xenobiotic metabolism, drug ADME (Absorption, Distribution, Metabolism, Excretion), lipid metabolism, and cytochrome enzymes (**Figure S1J-N, Figure S2A-F**). Furthermore, lipid metabolism, drug metabolism, and lipid digestion/absorption were significantly enriched in the organoids. Differential metabolite analysis via OPLS-DA revealed upregulation of glycolysis and lipid metabolism in the organoids, with specific metabolites such as xylitol, 1-octen-3-yl glucoside, and cortisol being more abundant in the organoids compared to 2D cultures (**Figure S1O&P, Figure S2G-H**). These results collectively demonstrate enhanced metabolism, biogenesis, and transport functions of hrHL after forming organoids.


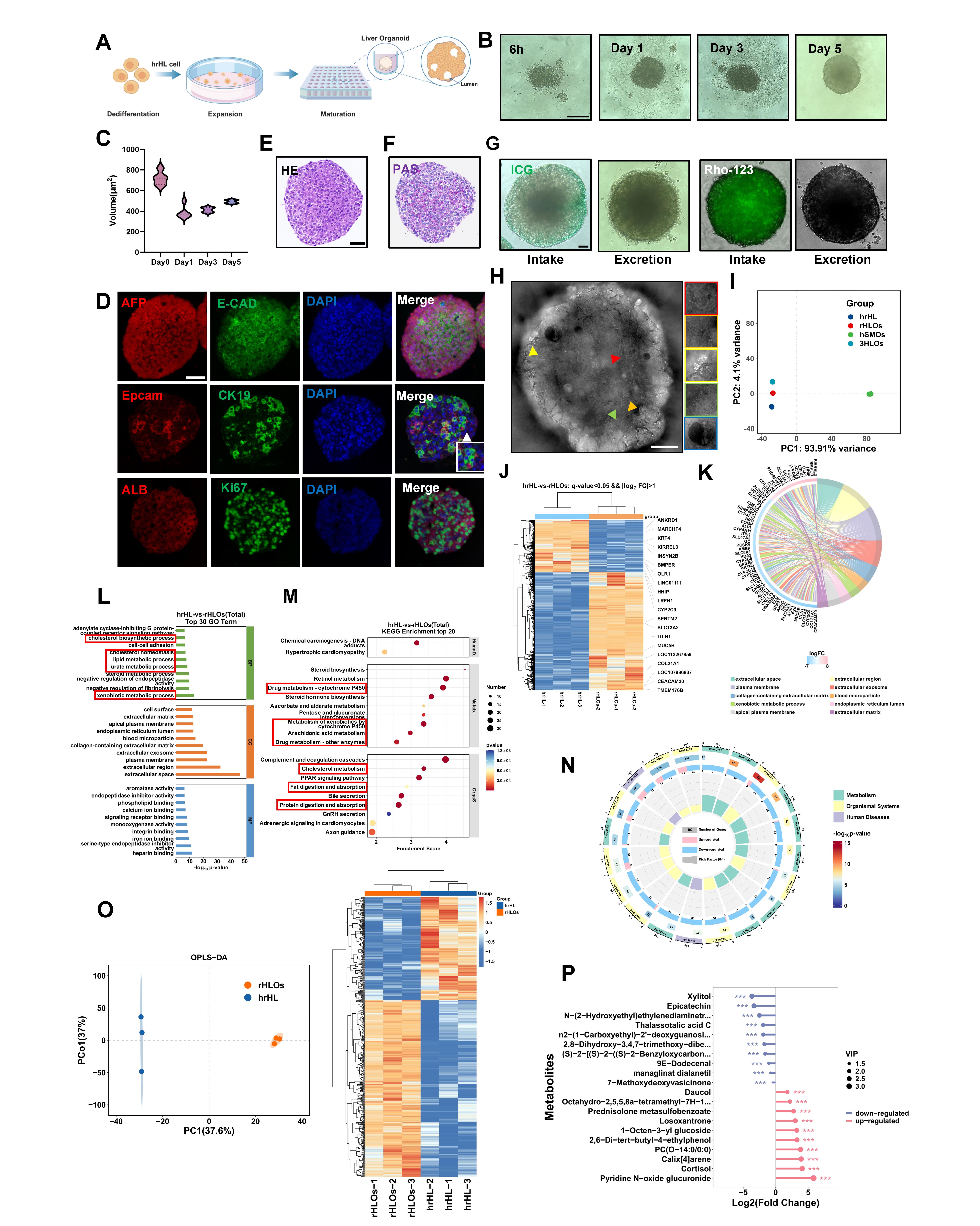


**Figure S1 rHLOs show multifaceted hepatic functions**

**A.** Schematic illustration of rHLOs formation from single hrHL cells and development of bile duct-like lumen structures within organoid.

**B-C**. Bright field images (**B**) and statistics of the projection area (**C**) of the organoids at 6h, and on 1, 2, 3, and 5 days post-hrHL seeding. (technical replicates ≥3, biological replicates ≥3, bold and narrow dash line indicated the median and 25th and 75th percentile, respectively). Scale bar: 50‌ μm.

**D.** Immunofluorescence staining of rHLOs to label AFP (fetal liver marker), E-CAD (epithelial marker), EpCAM & CK19 (intrahepatic bile duct markers), ALB (liver synthetic function marker), and Ki67 (proliferation marker). The white arrow in the second row indicates EpCAM & CK19 positive lumen-like structure. Scale bar: 50‌ μm

**E**. HE staining of rHLOs. White areas within the organoid indicate lumen-like structures. Scale bar:100‌ μm.

**F**. PAS staining of rHLOs. Positive areas (deep magenta) indicate glycogen storage within the 3HLOs. Scale bar:100‌ μm.

**G**. Intake and excretion assay of Indocyanine green and Rho-123 by rHLOs. Fluorescence (green) intensity indicates the quantity of the dye being uptake. Scale bar: 50‌ μm.

**H.** Visualization of the structures within the rHLOs with holographic digital microscopy. Structures being indicated with arrows and zoom-in in the right column indicate cell nuclei (red), mitochondria (orange), lysosomes (yellow), lipid droplets (green), and bile duct-like structures (blue). Scale bar: 50‌ μm.

**I-M**. PCA (**I**), heatmap (**J**), chord diagram (**K**), GO analysis (**L**), enrichment pathway analysis (**M**), and concentric circle analysis (**N**) of the differential transcriptome of rHLOs v.s. hrHL 2D cells. Drug, lipid, and cholesterol metabolism were enriched in (**M**) and analysis of liver metabolic function was focused in (**N**). n=3.

**O**. OPLS-DA analysis (left) and heatmap analysis (right) of metabolomic analysis between rHLOs and hrHL 2D cells. n=3.

**P**. Forest plot of metabolic changes analysis between rHLOs and hrHL 2D cells. n=3.


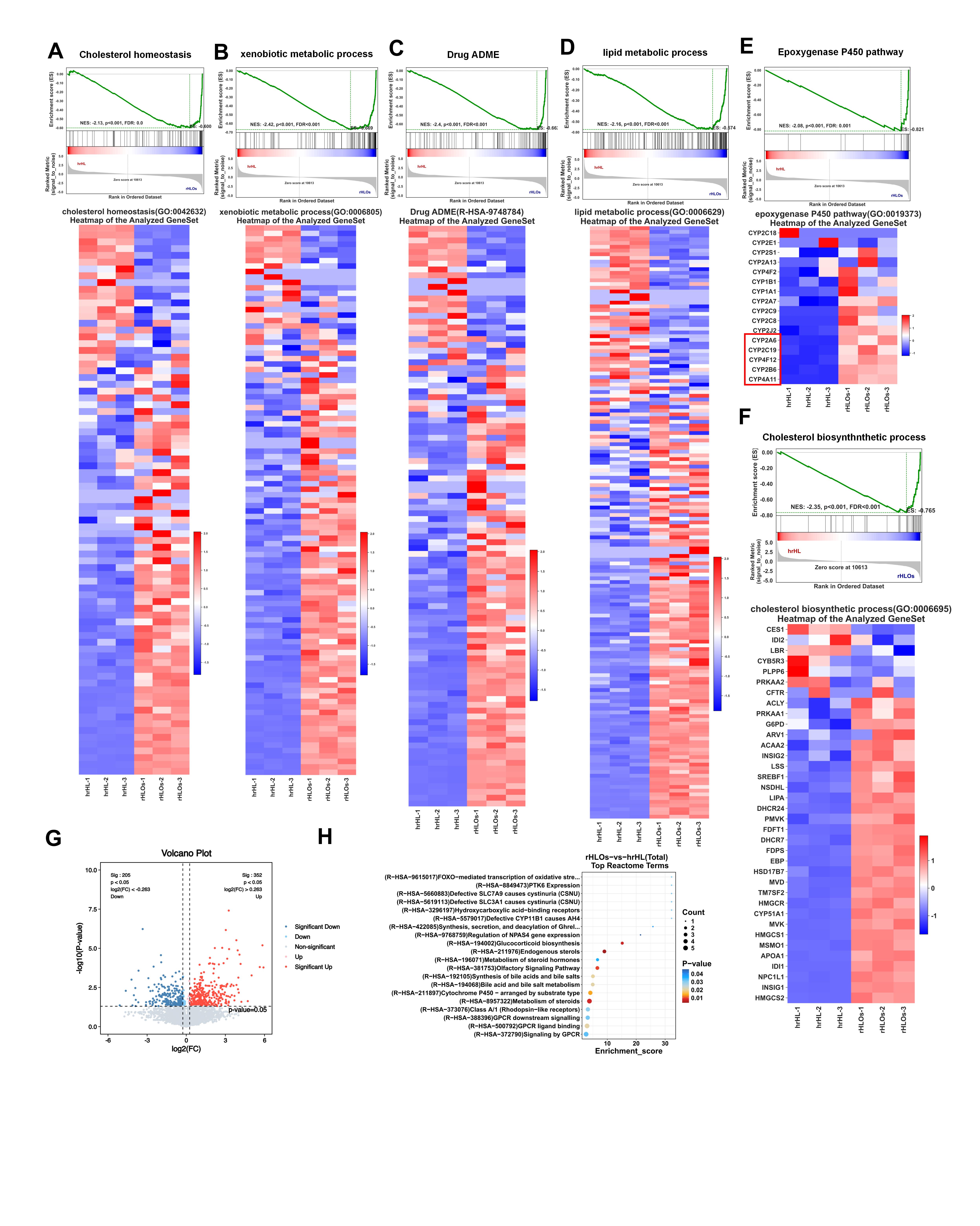


**Figure S2 Transcriptomic and Metabolomic Analysis of rHLOs and 2D Cell Lines.**

**A-F.** Heatmap analysis of rHLOs versus 2D cell lines: a. Heatmap of cholesterol homeostasis; b. Heatmap of xenobiotic metabolic process; c. Heatmap of drug ADME; d. Heatmap of lipid metabolic process; e. Heatmap of peroxygenase P450 pathway; f. Heatmap of cholesterol biosynthetic process.

**G.** Volcano plot of metabolomic analysis between rHLOs and 2D cell lines.

**H.** Concentric circle diagram of metabolomic analysis, with major changes concentrated in cellular processes and metabolism, etc.


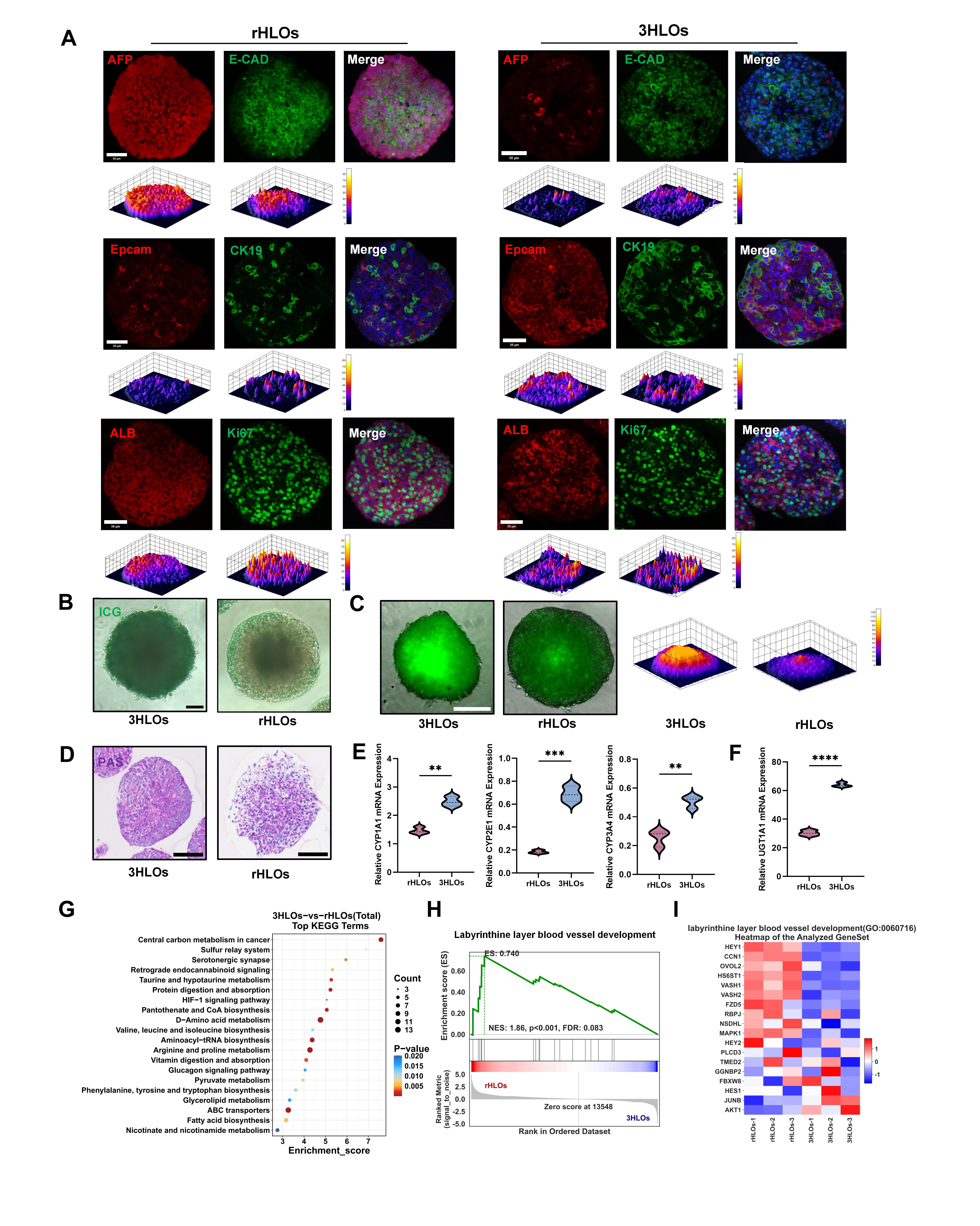


**Figure S3 Functional differences between 3HLOs and rHLOs.**

**A.** Analysis by IF staining, marking AFP (liver marker), E-CAD (epithelial marker), Epcam & CK19 (intrahepatic bile duct markers), Alb (liver function marker), and Ki67 (proliferation marker). Images captured using Leica SP8 optical instrumentation, followed by surface plot analysis using ImageJ to reflect expression changes of corresponding markers. Scale bar:50 ‌μm

**B.** Comparison of ICG uptake between the two types of organoids, highlighting uptake differences. Scale bar:50 μm

**C.** Comparison of Rho-123 uptake between the two types of organoids, with surface plot analysis using ImageJ to analyze Rho-123 distribution. Scale bar:50 μm

**D.** PAS staining analysis of the differences in glycogen storage between the two types of organoids. Scale bar:50 μm

**E.** Detection of CYP1A1, CYP2E1, and CYP3A4 in the P450 enzyme family by qPCR. n=5. ** *P* < 0.01; *** *P* < 0.001.

**F.** Detection of metabolic enzyme UGT1A1 expression by qPCR.n=5. **** *P* < 0.0001

**G.** Comparative KEGG pathway analysis of 3HLOs and rHLOs revealed distinct functional enrichments.

**H&I.** Heatmap analysis of labyrinthine layer blood vessel development, showing that inhibitory transcription factors for angiogenesis (HEY1, CCN1, VASH1/2) are expressed in 3HLOs.

**
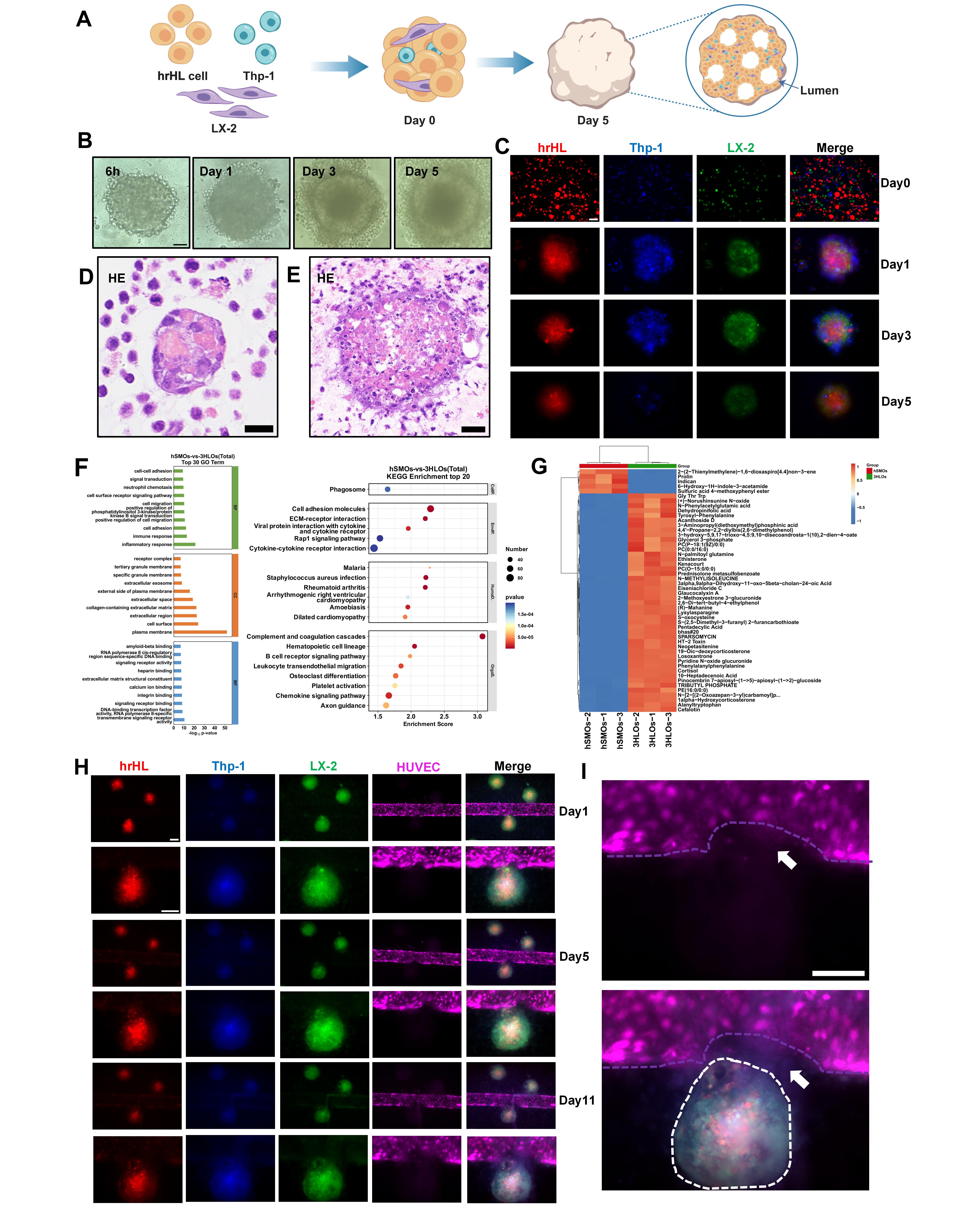
**

**Figure S4 On the construction and functional characterization of hSMOs.**

**A.** Construction process of hSMOs, composed of hrHL cells, Thp-1 cells, and LX-2 cells, with distinct vacuolar structures formed internally after maturation.

**B.** Observation and recording of growth changes in hSMOs. Scale bar:50 ‌μm

**C.** Observation of the formation process of hSMOs using hrHL (red), Thp-1 (blue), and hepatic stellate cells (LX-2). Time from Day 1 to Day 5. Scale bar:50 ‌μm

**D.** HE staining analysis of hSMOs, revealing scattered cell distribution externally.

**E.** Analysis of hSMOs in the microfluidic chip, showing distinct scattered cell distribution, less effective than rHLOs and 3HLOs.

**F.** Transcriptomic sequencing analysis of hSMOs and 3HLOs, with GO analysis focused on changes in immune and inflammatory responses (left). Signal pathway analysis mainly concentrated on interactive responses of cytokines (right).

**G.** Metabolomic analysis of changes between hSMOs and 3HLOs, finding major changes concentrated in 3HLOs.

**H.** Observing changes in hSMOs (hrHL cells-red, Thp-1 cells-blue, LX-2 cells-green) relative to main channel blood vessels (purple) from Day 1 to Day 11. Scale bar:50 ‌μm

**I.** Observing that hSMOs erode the main channel blood vessels, indicated by white arrows. Scale bar:50 ‌μm


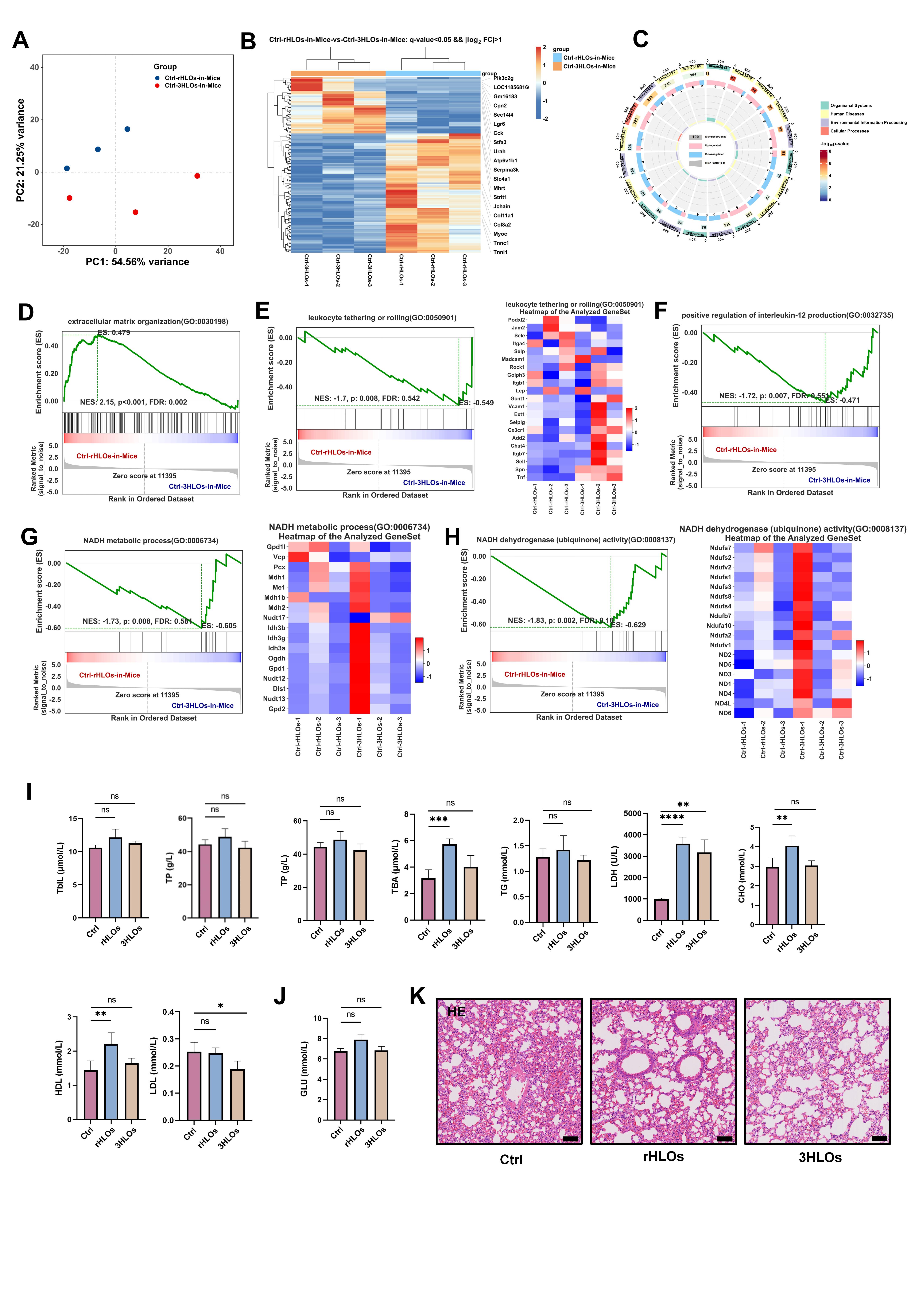


**Figure S5 Transcriptome analysis of subcutaneously loaded rHLOs and 3HLOs and safety testing.**

1. PCA results of transcriptome analysis of in vivo transplanted 3HLOs and rHLOs.
2. Top significant DEG of 3HLOs and rHLOs.
3. Concentricity analysis of 3HLOs and rHLOs.
4. GSEA diagrams of both organoids regarding extracellular matrix.
5. GSEA diagram of rolling of leukocytes (left). GSEA diagrams of leukocytes regarding extracellular matrix. GSEA diagram of leukocyte rolling (right). It mainly includes biological process, cellular component, and molecular function.
6. Analysis of GSEA regarding the pathway of IL-12 production activation in both organoids.
7. GSEA graph (left) and heat map analysis (right) regarding the process of NADH metabolism, with elevated expression of Nudt17 in 3HLOs.
8. GSEA (left) and heat map (right) regarding the activation of NADH dehydrogenase, with elevated expression of ND4L and ND6 in 3HLOs.
9. Serologic examination of liver function and other function in the Ctrl group, rHLOs group, and 3HLOs group.n=5. ns *P* > 0.05; * *P* < 0.05; ** *P* < 0.01; *** *P* < 0.001; **** *P* < 0.0001.
10. Serologic examination of GLU in the Ctrl group, rHLOs group, and 3HLOs group. ns *P* > 0.05.
11. Changes in the lungs of nude mice in the Ctrl, rHLOs, and 3HLOs groups were analyzed by HE staining. Scale bar:50 μm.


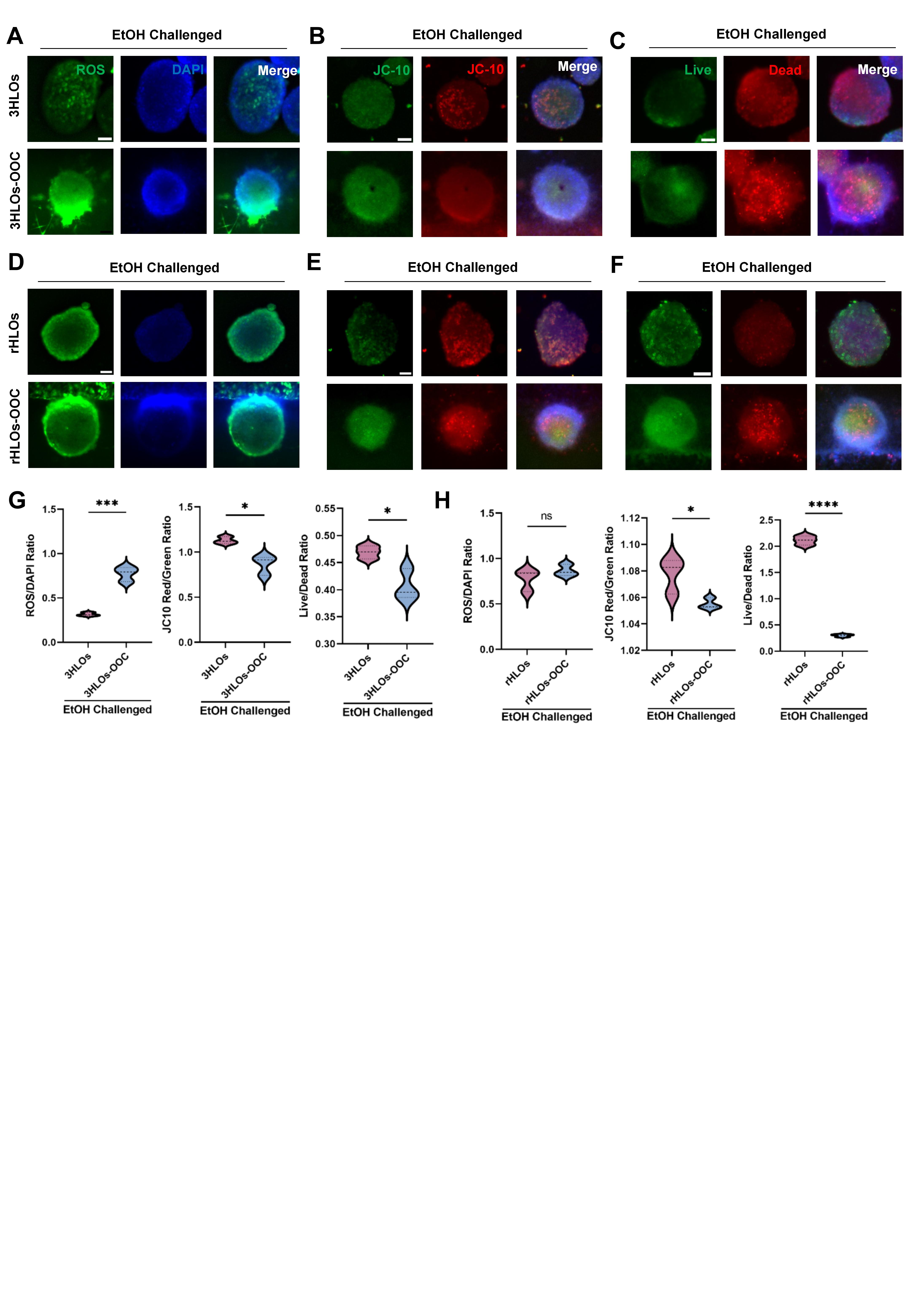


**Figure S6 Comparative Analysis of Organoids-on-Chips and Organoids in ALI Modeling**

1. **C.** Observations ALI model of ROS, JC-10 and viability changes in 3HLOs and 3HLOs-OOC.Scale bar:50 μm.
2. **F**. Observations ALI model of ROS, JC-10 and viability changes in rHLOs and rHLOs-OOC.Scale bar:50 μm.

**G**-**H**. ImageJ analysis of ROS/DAPI ratio (left), JC-10Red/JC-10Green ratio (middle), and Live-Green/Dead-Red cell ratio (right) changes. ns *P* >0.05; * *P* < 0.05; *** *P <* 0.001; **** *P <* 0.0001.


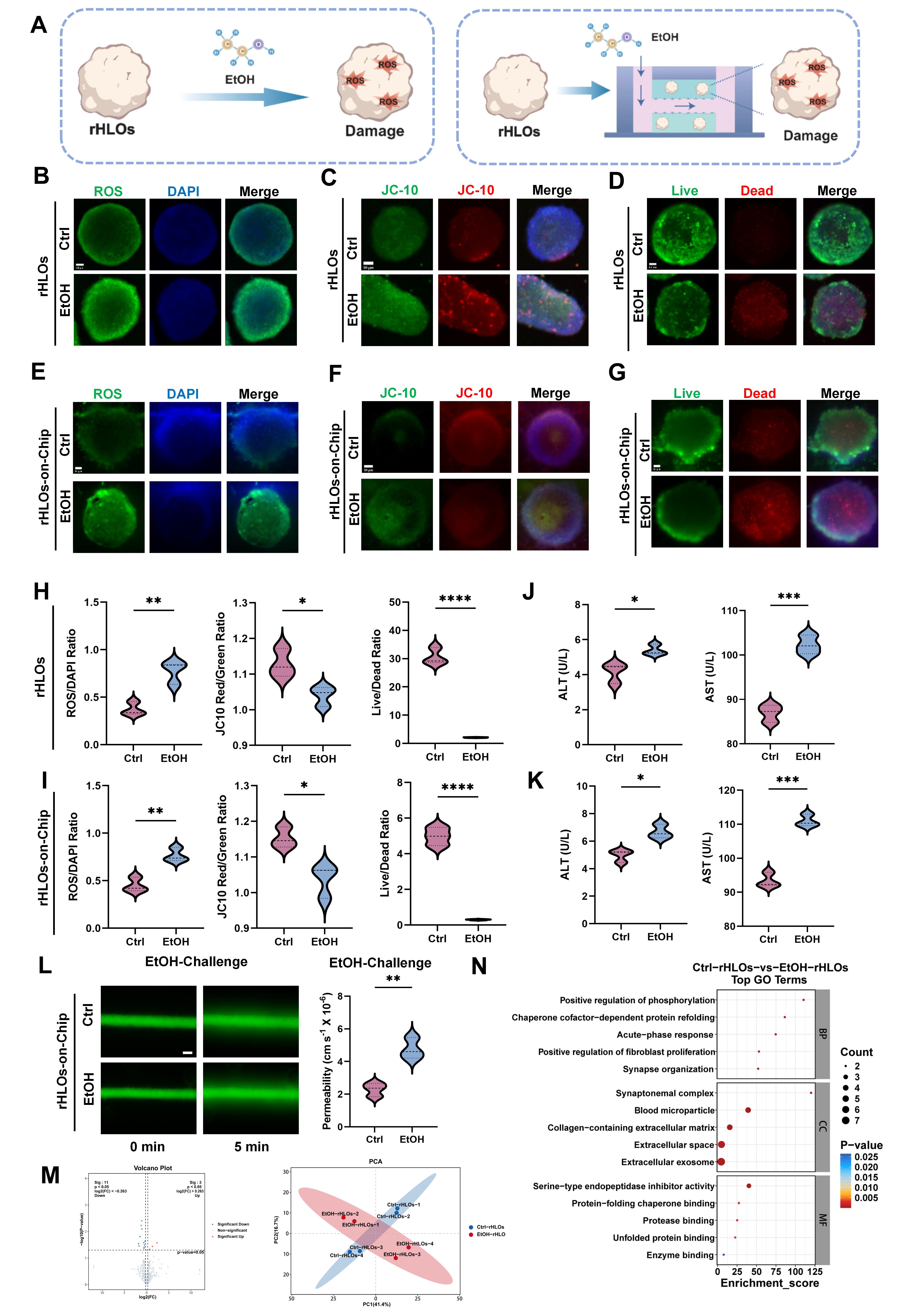


**Figure S7 Construction of Alcoholic Liver Disease Model Using rHLOs and Microfluidic Chip Technology.**

**A.** Direct construction of an alcoholic liver disease model using rHLOs (left); combination of organoid technology and microfluidic chip technology to construct a highly biomimetic organ chip for alcoholic liver disease model (right).

**B.** Observations of ROS changes in Ctrl group, EtOH group, with ROS staining (green) followed by imaging with Echo optical microscopy. Scale bar:50 μm.

**C.** Changes in JC-10 in Ctrl group, EtOH group, with JC-10 staining (JC-10 red/green) followed by imaging with Echo optical microscopy. Scale bar:50 μm.

**D.** Observations of viability changes in Ctrl group, EtOH group, with live/dead staining (live cells: green, dead cells: red) followed by imaging with Echo optical microscopy. Scale bar: 50‌ μm.

**E-G.** After constructing rHLOs chip, establishing the alcoholic liver disease model. Detecting ROS (E), JC-10 (F), and viability (G) changes in rHLOs within the chip. Scale bar: 50 μm.

**H&I.** ImageJ analysis of ROS/DAPI ratio (left), JC-10Red/JC-10Green ratio (middle), and Live-Green/Dead-Red cell ratio (right) changes in Ctrl group, EtOH group. h: rHLOs modeling; i: rHLOs modeling in microfluidic chip. n=12. * *P* < 0.05; ** *P* < 0.01; *** *P* < 0.001; **** *P* < 0.0001.

**J.** Detection of ALT and AST in the supernatant of rHLOs, comparing changes among Ctrl group, EtOH group. n=5. ns *P* > 0.05; * *P* <0 .05.

**K.** Detection of ALT and AST in the supernatant of rHLOs in the microfluidic chip, comparing changes among Ctrl group, EtOH group. n=5. ns *P* > 0.05; * *P* < 0.05.

**L.** Observing the tightness of blood vessels in Ctrl group, EtOH group after injecting 70kDa into the main channel blood vessel. Calculating 70kDa penetration using the Poisson equation. n=5. ns *P* >0.05; * *P* < 0.05. Scale bar:50 μm.

**M-N.** PCA (M) and GO (N) protein metabolism analysis in EtOH and Ctrl groups.

**Table S1. Antibody Information**

| **Antibody Name** | **Company** | **Catalog No.** |
| --- | --- | --- |
| Afp | Abcam | ab169552 |
| Alb | Abcam | ab207327 |
| E-cad | Abcam | ab287970 |
| CK19 | Abcam | ab76539 |
| EpCAM | Abcam | ab223582 |
| CD31 | Abcam | ab28364 |
| MMP3 | Abcam | ab194717 |
| Ki67 | Abcam | ab279653 |
| hCD31 | Dako | M0823 |
| Pdgfrβ | Abcam | Ab196376 |

**Table S2.** **Primer sequences**

| **Gene Name** | **F/R** | **Nucleobase Sequence** |
| --- | --- | --- |
| *CYP1A2* | Forw6ard primer | CTGGGCACTTCGACCCTTAC |
|  | Reverse primer | TCTCATCGCTACTCTCAGGGA |
| *CYP3A4* | Forward primer | AAGTCGCCTCGAAGATACACA |
|  | Reverse primer | AAGGAGAGAACACTGCTCGTG |
| *CYP2E1* | Forward primer | ATGTCTGCCCTCGGAGTCA |
|  | Reverse primer | CGATGATGGGAAGCGGGAAA |
| *UGT1A1* | Forward primer | CATGCTGGGAAGATACTGTTGAT |
|  | Reverse primer | GCCCGAGACTAACAAAAGACTCT |
| *GAPDH* | Forward primer | GGAGCGAGATCCCTCCAAAAT |
|  | Reverse primer | GGCTGTTGTCATACTTCTCATGG |
| *TNF* | Forward primer | CCCTCACACTCAGATCATCTTCT |
|  | Reverse primer | GCTACGACGTGGGCTACAG |
| *CCL4* | Forward primer | TTCCTGCTGTTTCTCTTACACCT |
|  | Reverse primer | CTGTCTGCCTCTTTTGGTCAG |
| *CCL2* | Forward primer | TTAAAAACCTGGATCGGAACCAA |
|  | Reverse primer | GCATTAGCTTCAGATTTACGGGT |

**Reference**

[1] P. Y. Huang, L. D. Zhang, Y. M. Gao, Z. Y. He, D. Yao, Z. T. Wu, J. Cen, X. T. Chen, C. C. Liu, Y. P. Hu, D. M. Lai, Z. L. Hu, L. Chen, Y. Zhang, X. Cheng, X. J. Ma, G. Y. Pan, X. Wang, L. J. Hui, *Cell Stem Cell* **2014**, 14, 370.

[2] X. Yuan, J. Q. Wu, Z. Sun, J. Cen, Y. J. Shu, C. H. Wang, H. Li, D. N. Lin, K. Zhang, B. H. Wu, A. Dhawan, L. D. Zhang, L. J. Hui, *Cell Stem Cell* **2024**, 31.
